# Virtually isolated: social identity threat predicts social approach motivation via sense of belonging in computer-supported collaborative learning

**DOI:** 10.3389/fpsyg.2024.1346503

**Published:** 2024-09-13

**Authors:** Nathalie Bick, Laura Froehlich, Jan-Bennet Voltmer, Jennifer Raimann, Natalia Reich-Stiebert, Niels Seidel, Marc Burchart, Sarah E. Martiny, Jana Nikitin, Stefan Stürmer, Andreas Martin

**Affiliations:** ^1^CATALPA – Center of Advanced Technology for Assisted Learning and Predictive Analytics, FernUniversität in Hagen, Hagen, Germany; ^2^Department of Psychology, FernUniversität in Hagen, Hagen, Germany; ^3^Department of Mathematics and Computer Science, FernUniversität in Hagen, Hagen, Germany; ^4^Research Institute for Telecommunications and Cooperation e.V., Dortmund, Germany; ^5^Department of Psychology, UiT The Arctic University of Norway, Tromsø, Norway; ^6^Department of Developmental and Educational Psychology, University of Vienna, Vienna, Austria; ^7^Department of Humanities and Social Sciences, FernUniversität in Hagen, Hagen, Germany; ^8^German Institute for Adult Education – Leibniz Centre for Lifelong Learning, Bonn, Germany

**Keywords:** behavioral data, higher distance education, collaborative writing, social psychology, random intercept cross-lagged panel model

## Abstract

Collaboration improves multiple academic and social outcomes. Accordingly, computer-supported collaborative learning (CSCL) can be beneficial in distance education contexts to overcome the issues specific to online learning (e.g., underperformance, low identification with university). Distance universities often attract a substantial number of non-traditional students (e.g., students with disability, students with migration background). Despite their representation, non-traditional students face negative stereotypes and associated social consequences, including social identity threat, diminished sense of belonging, and less motivation for social interactions. In the context of online learning, where there is little individuating information, social categories like socio-demographic group memberships become salient, activating stereotypes. Consequently, socio-demographic group memberships can have detrimental consequences for the integration of non-traditional students. The purpose of the present study was to (a) determine the extent of social identity threat for students in higher distance education, (b) explore the social consequences of this threat in the same context, (c) validate these findings through longitudinal analyses embedded in a CSCL task, and (d) use learning analytics to test behavioral outcomes. In a longitudinal study with three measurement occasions over 8 weeks (*N* = 1,210), we conducted path analyses for cross-sectional associations and Random Intercept Cross-Lagged Panel Models for longitudinal predictions. The results showed that non-traditional students mostly reported higher social identity threat than traditional students. While the expected longitudinal within-person effects could not be demonstrated, we found stable between-person effects: students who reported higher levels of social identity threat also reported lower sense of belonging and lower social approach motivation. Exploratory analyses of actual online collaboration during CSCL offer potential avenues for future research. We conclude that social identity threat and its social consequences play an important role in higher distance education and should therefore be considered for successful CSCL.

## 1 Introduction

Underperformance, dropout, and low identification with the university are commonly discussed issues in higher distance education (e.g., Stoessel et al., 2015). Computer-supported collaborative learning (CSCL) is a promising format to address these issues (Kirschner et al., 2004). Collaborative learning formats are associated with positive academic and social outcomes, such as improved performance and class attendance, student familiarity with the faculty, and understanding of diversity (Panitz, 1999; Roberts, 2005). CSCL uses technology to improve learning in the context of reading and writing tasks (Stahl et al., 2006) through collaboration. Research has shown that the key aspect of collaboration, namely social learning (Roschelle and Teasley, 1995), is associated with improved academic achievement (Talan, 2021), more frequent interactions with peers (Shin et al., 2020), and problem-solving skills. CSCL thus has the potential to address the described issues related to academic and social outcomes in higher distance education.

The high degree of temporal and spatial flexibility in higher distance education leads to a heterogeneous student body and an overrepresentation of non-traditional students at distance education institutions (i.e., students from sociodemographic groups who have been underrepresented in higher education in the past; e.g., students with disability, students with migration background; Schneller and Holmberg, 2014; Stoessel et al., 2015). However, non-traditional students are particularly at risk of underachievement and dropping out of higher education (Stoessel et al., 2015). Therefore, we argue that given the above-mentioned advantages, digital collaborative learning can be beneficial especially for non-traditional students. Moreover, the reduction of performance gaps between traditional and non-traditional students is an important goal especially at higher distance education institutions (Stoessel et al., 2015) and could be motivated by diversity being associated with several advantages for all students, e.g., increased intercultural competencies, understanding, and empathy, better preparation for employment in the global economy, or increased engagement in political issues and participation in democratic processes (Wells et al., 2016). Furthermore, the financial and reputation-related losses due to student dropout from the universities’ perspective (Raisman, 2013) might contribute to the motivation to reduce academic underperformance of specific groups.

### 1.1 Social identity threat as a potential risk in higher distance education

Despite the advantages of higher distance education for non-traditional students and the potential benefits of CSCL described above, social-psychological research has identified risks of increased stereotyping and associated negative consequences for non-traditional students during computer-mediated communication. In the current research we investigate CSCL in the form of a collaborative writing task spanning several weeks. This type of CSCL predominantly involves asynchronous computer-mediated communication, where information about individual traits and characteristics of a person (i.e., individuating information) is less frequent than information about social category memberships (e.g., age, gender, ethnicity). Social categories are more salient compared to face-to-face contexts (Postmes et al., 2001; Spears et al., 2002) and we therefore assume that stereotypes associated with social categories are prone to be activated in CSCL as investigated in the current research.

The diverse student body in higher distance education includes numerous student groups that are stereotypically associated with low academic competence (e.g., students with chronic illness, students with non-German native language; Bick et al., 2022). These student groups are thus at risk of experiencing detrimental consequences when negative competence-related stereotypes are activated. One prominent consequence of stereotype activation is social identity threat (Steele and Aronson, 1995; Schmader et al., 2008; Spencer et al., 2016). According to social identity theory (Tajfel and Turner, 1979), people strive for a positive social identity (i.e., a positive differentiation or distinction of one’s own group from other groups). Negative stereotypes threaten this positive social identity, which leads to impairments in various domains. For example, as second-language learners are often stereotypically associated with low verbal competence, a student with non-native language might worry that their contributions in a collaborative writing task might be negatively evaluated by others in the CSCL group. This worry in turn makes it more difficult for them to show their full intellectual potential and feel like they fit in at university and the CSCL group. A large number of studies has shown performance-related consequences of social identity threat in face-to-face contexts, e.g., for women (Schmader, 2002; Bell et al., 2003), older people (Hess et al., 2003; Lamont et al., 2015), or immigrants (Steele and Aronson, 1995; Appel et al., 2015). However, to our knowledge a systematic investigation of social identity threat in higher distance education is still lacking. Additionally, a first study about widespread stereotypes about student groups in distance education showed that stereotypes about student groups are to some extent specific to the context of higher distance education (e.g., positive evaluation of older students; Bick et al., 2022). Therefore, the first aim of the current research is to investigate the extent of social identity threat experienced by students identifying with different sociodemographic student groups at a large German distance university.

### 1.2 Consequences of social identity threat for social relationships

In addition to the well-investigated performance-related consequences of social identity threat, a growing body of research focuses on social consequences of social identity threat (Good et al., 2012; Martiny and Nikitin, 2019; Rahn et al., 2021; Froehlich et al., 2022). As CSCL is inherently a social activity, the second aim of the current research is therefore to investigate these social consequences of social identity threat in higher distance education. One consequence is that people disengage with the academic field in which the negative stereotype occurs and no longer identify with it (Steele et al., 2002; Woodcock et al., 2012). Accordingly, disidentification from academia is most common among students who face negative stereotypes in higher distance education, i.e., non-traditional students (e.g., Gopalan and Brady, 2020). One aspect of disidentification is the questioning of one’s social ties and the feeling of not “fitting in” (i.e., belonging uncertainty; Walton and Cohen, 2007). A lower sense of belonging to academia is in turn associated with academic disadvantage (Mahoney and Cairns, 1997; Walton and Cohen, 2011; Good et al., 2012) as well as lower engagement for studying and lower intention to stay at university, particularly for ethnic minority students (Zea et al., 1997; Just, 1999; Wolf et al., 2021). Non-traditional students might therefore be disadvantaged due to a lack of social connectedness (Yildirim et al., 2021).

Most research on sense of belonging in the educational domain focuses on students’ sense of belonging to academia in general or to their academic institution (e.g., Meeuwisse et al., 2010; Abdollahi and Noltemeyer, 2018; Martiny and Nikitin, 2019). Differentiating specific domains to which students report they belong led to a more accurate understanding of which domain is relevant to students in different contexts (e.g., Good et al., 2012). In higher distance education, students might only weakly identify with the university as they rarely visit the campus. When students collaborate in virtual study groups, their sense of belonging to the study group might in fact be more important than their sense of belonging to the university. Research with high-school students in face-to-face education has shown different findings with regard to sense of belonging to the class or school (Froehlich et al., 2023). Therefore, in the present research we assess sense of belonging to the CSCL group and to the university. We expect sense of belonging to the CSCL group to be more closely linked to social identity threat and our outcome variables related to social relationships. We also investigate whether the results with sense of belonging to the university are comparable to the results with sense of belonging to the CSCL group.

The main outcome variables in the current research are students’ motivation and behavioral tendencies to form peer relationships and their collaboration behavior during the CSCL task. We base our predictions for these outcome variables on previous research conducted in face-to-face learning contexts, extend the research focus to computer-mediated communication, and use learning analytics to shed light on the potential social consequences of social identity threat in CSCL. In studies with immigrant students in Norway and Germany, students who reported higher social identity threat also reported lower sense of belonging (Froehlich et al., 2022, 2023). In addition, these studies examined sense of belonging as a mediator between social identity threat and social approach motivation, as well as behavioral intentions for contact as outcome measures for social connectedness. Social approach motivation (i.e., the motivation to initiate and maintain social relationships; Elliot et al., 2006) is associated with less loneliness and more satisfaction with social ties (Gable, 2006), which in turn is associated with physical health and subjective well-being (Hawkley and Cacioppo, 2010; Nikitin et al., 2012). Especially for non-traditional students who are struggling with family obligations or chronic illness, or who have difficulties in accessing information due to language difficulties, social approach motivation in educational contexts might play a crucial role, as social relationships at university bring study-related advantages (e.g., Ma and Yuen, 2011; Raaper et al., 2022).

### 1.3 Methodological advancements when investigating social identity threat in distance education: longitudinal analysis and learning analytics

Previous research has found that sense of belonging mediates the relationship between social identity threat and social approach motivation among participants with different social identities, of different age, and in different European countries (Martiny and Nikitin, 2019; Rahn et al., 2021; Froehlich et al., 2022, 2023). The present study aims to replicate these findings with heterogeneous student groups in higher distance education and thus to corroborate the generalizability of the effect. Moreover, the social consequences of social identity threat have so far mainly been investigated in cross-sectional studies which provide only limited evidence on the directionality of the effects. The third aim of the present research is therefore to investigate the meditation of social identity threat and social approach motivation by sense of belonging in a longitudinal design to test the proposed directionality of effects. CSCL tasks often involve collaboration over several weeks during the semester so that multiple measurement occasions can be integrated into the online learning environment. A longitudinal design allows us to test whether associations can be replicated at the level of individual students (within-person effects), while the between-person effects are statistically accounted for in a Random Intercept Cross-Lagged Panel Model (RI-CLPM; Hamaker et al., 2015). Thus, within- and between-person effects that have been confounded in previous cross-sectional studies can be disentangled to investigate whether the hypothesized associations of social identity threat, sense of belonging, and social outcomes are between-person (i.e., reflecting a stable rank order between students in classrooms or distance education courses) or within-person (i.e., reflecting psychological processes that unfold over time at the intraindividual level).

The fourth and final aim of the present research is to expand the outcome variables concerning the social consequences of social identity threat and make use of behavioral data available through learning analytics in the context of distance education. To complement previous research in face-to-face contexts (Froehlich et al., 2022), we assessed the same self-report outcome variables of social approach motivation and behavioral intentions for contact with other students. The assessment of behavioral intentions is common in social psychology because the measurement of actual behavior is difficult and resource-intensive in many traditional study designs. However, there is usually a gap between intentions and actual behavior (Webb and Sheeran, 2006). The mainly asynchronous CSCL context of the present study, in which students’ behavior is stored in databases and logfiles, provided a unique opportunity to combine established self-report measures with measures of student behavior in virtual groups using collaborative distance learning tools. To this end, we used data from students’ interactions around a collaborative writing task to conduct social network analysis directly reflecting the social relationships between students. These data refer to the group communication and coordination in a course-related Moodle forum and the actual shared writing process in a real-time collaborative text editor.

### 1.4 The present research and hypotheses

Based on the considerations outlined above, we argue that low social identity threat, high sense of belonging, and high social approach motivation are important prerequisites to successful CSCL. Furthermore, we are convinced that collaboration can be especially beneficial for non-traditional students who are overrepresented in higher distance education. Thus, the higher distance education context is very suitable for the investigation of the role of social identity threat and sense of belonging for social approach motivation and peer relationships. Accordingly, the Hypotheses of the present study are threefold. First, we descriptively investigate perceived social identity threat for different student groups to identify groups who are particularly at risk for social identity threat in higher distance education. Second, we expect to replicate the cross-sectional findings on the relationship between social identity threat and social approach motivation via sense of belonging, previously shown in face-to-face contexts (e.g., Martiny and Nikitin, 2019; Froehlich et al., 2022, 2023), in the context of higher distance education. Third, we apply data-driven methods in a longitudinal field study to validate self-report findings with data from actual learner interactions over time. We apply a learning analytics approach combining self-report data and behavioral data. During a CSCL task spanning several weeks, we collected self-report data at multiple occasions. In addition, we collected fine-grained process data (e.g., Moodle forum activity, writing/ deleting/ copying/ pasting/ formatting text) of learning activities taking place within the online learning environment. At the beginning of the semester, students participated in a demographic survey (T0). After being assigned to the CSCL groups and a phase of getting to know each other, students participated in the first survey (T1). This was followed by two working phases of the CSCL task with an interim survey (T2) and a final survey (T3).

**Figure 1:**
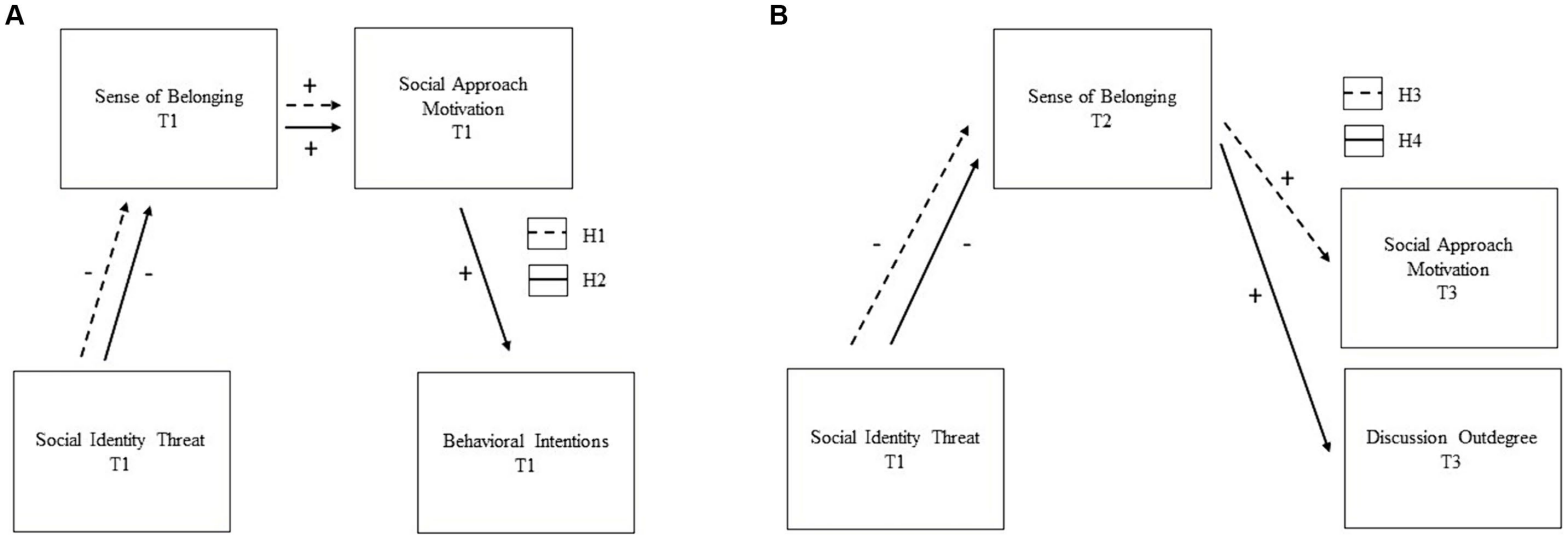
Expectations regarding cross-sectional **(A)** and longitudinal **(B)** Hypotheses. All variables depicted in **(B)** were assessed before/ at T1, T2, and T3. To reduce complexity, only the time points relevant to our hypotheses are presented. The corresponding RI-CLPM are shown in Figures 4, 5.

We investigated the following pre-registered Research Question (RQ) and Hypotheses (H^1^) which are depicted in Figure 1: We descriptively investigated perceived social identity threat for different student groups at different measurement occasions (RQ). We hypothesize a simple cross-sectional mediation with higher social identity threat negatively predicting self-reported social approach motivation via lower sense of belonging at T1 (H1). Further, we hypothesize a serial cross-sectional mediation with social identity threat as the predictor, sense of belonging and social approach motivation as mediators, and self-reported behavioral intentions as outcome at T1 (H2). Third, in a simple longitudinal mediation model, we expect higher social identity threat at T1 to negatively predict social approach motivation at T3 via lower sense of belonging at T2 (H3). Furthermore, in another longitudinal mediation model, we expect that higher social identity threat at T1 negatively predicts the behavioral measure of the individuals’ integration in the CSCL group (i.e., discussion outdegree) during the final cooperation phase (between T2 and T3) via lower sense of belonging at T2 (H4).

## 2 Materials and methods

The study was conducted in accordance with open science principles (pre-registration of Hypotheses, open materials, open data). The pre-registration, materials, data, and script can be found on the OSF: https://osf.io/axq7k/.

### 2.1 Participants and data collection

Data collection for this study was embedded in a superordinate project implemented in a mandatory course of the introductory module of the Bachelor’s degree program in psychology at a large distance university. The beginning of the Bachelor’s degree is an important academic transition to higher distance education. In addition, it provides the best opportunity to investigate social identity threat and its consequences, as the dropout rate is highest in the first two semesters when the introductory module takes place (Neugebauer et al., 2019). During the course, students interacted in a course-related Moodle forum. The longitudinal design consisted of three voluntary measurement occasions with self-report questionnaires in Unipark^2^ with two- or three-week intervals. At the beginning of the semester, students completed a demographic survey (T0). They were then assigned to CSCL groups of eight and had 2 weeks to get to know each other. The first main measurement (T1) was conducted before the first CSCL phase started. The first CSCL phase consisted of 3 weeks in which students collaborated to summarize the introduction and the methods sections of a peer-reviewed journal article. The second measurement (T2) was conducted afterwards. The subsequent second CSCL phase again consisted of 3 weeks in which the results and discussion sections of the same article were summarized, followed by the final measurement (T3). Both CSCL tasks were completed online in individual Etherpad Lite instances^3^^,^^4^ for each CSCL group which were provided by a Collaborative Learning Platform to support collaborative writing in large-scale distance education (Burchart and Haake, 2023). Each CSCL group was provided with an individual Etherpad Lite instance containing only the Etherpad documents (Pads) of the particular group. Therefore, the actual collaboration took place during the working phases in the shared text documents of the group, whereas the Moodle forum served as a communication platform during all three phases. The self-report measures (i.e., group identification, social identity threat, sense of belonging, social approach motivation, behavioral intentions for contact) were presented in the final section of the superordinate project’s survey at all three measurement occasions (T1–T3) and the demographic survey (T0).However, self-report measures at T0 were incomplete (i.e., only group identification, social identity threat, and sense of belonging to the university assessed) due to a programming issue. Students were informed about data protection, the content and duration of the survey, and provided written consent for participation according to EU General Data Protection Regulations and research ethics guidelines by the American Psychological Association (APA), the German Psychological Association (DGPs), and the Declaration of Helsinki (World Medical Association Declaration of Helsinki, 2013). Within each main measurement occasion, students started with indicating which student group(s) they identified with. Next, items on sense of belonging to the university and the CSCL group, social approach motivation, and behavioral intentions for contact were completed. Finally, the students provided written consent to the scientific use of their data. To match data from the surveys, students generated an individual pseudonymized code. Survey data were matched with behavioral data from Moodle and Etherpad Lite by an independent data trustee. Since data were collected using three different systems (Unipark, Moodle, Etherpad Lite) students were assigned to individual identifiers in each system. It was ensured that none of the researchers involved had technical access to more than one of these three systems. The data trustee replaced the respective identifiers of the individual systems with a unique key for each student. The unique key consisting of a 41-digit hexadecimal code was generated using a hash function including a secret salt. For ensuring data privacy requirements, this unique key can only be related to the identifiers of the three systems by the data trustee but not by the involved researchers, administrators, or teachers.

Data were collected from October to December 2022. Survey participation was compensated with course credit. We applied the following pre-registered exclusion criteria: After excluding participants who did not answer any of our main items (*n* = 631), did not consent to the scientific use of their data (*n* = 22), or did not identify with at least one of the student groups (*n* = 57), *N* = 1,210 undergraduate psychology students were included in the sample. Of those who completed the demographic survey at T0 (*n* = 694), 52.7% were under the age of 30 and 46.8% were 30 years or older. Furthermore, 69.8% indicated a female and 28.7% a male gender. Around a quarter (25.5%) stated that they have a migration background and 14.0% reported that their native language is not German. More than a third (35.3%) reported to be in full-time employment. Due to dropout during the course, the CSCL groups differed regarding their number of active students. On average, 4 to 5 of the 8 students assigned to each group actively contributed to the CSCL task, *M*_T1_ = 4.4, *SD*_T1_ = 1.3; *M*_T2_ = 4.8, *SD*_T2_ = 1.5; *M*_T3_ = 4.3 *SD*_T3_ = 1.3.

Sample size was determined by the number of students in the introductory module of the Bachelor’s degree program in psychology who voluntarily participated in the survey. We therefore relied on pre-registered rules of thumb to determine the statistical power for testing our Hypotheses. According to Pan et al. (2018, tbl. 5), our sample was sufficiently large (i.e., *N* > 544) for a longitudinal mediation analysis based on Bootstrap estimation with three measurement points, high intra-class correlation (ICC = 0.90), and small effects (i.e., *b* = 0.14) for the *a* and the *b* path. Note that we incorrectly referred to Table 4 (medium intra-class correlation; ICC = 0.60) instead of Table 5 (Pan et al., 2018) in the pre-registration, resulting in a smaller pre-registered sample size (*N* = 385). However, the achieved sample of *N* = 1,210 was larger than the required minimum sample sizes in both tables so the statistical power was sufficiently large to test the pre-registered Hypotheses.

### 2.2 Measures

#### 2.2.1 Self-report measures

As a measure of *group identification,* students indicated which sociodemographic student group(s) they identified with at university over the course of the semester. Ten student groups were presented based on previous research on stereotypes about student groups in higher distance education (Bick et al., 2022): female students (students who identify with the female gender), male students (students who identify with the male gender), students with chronic illness (students who have a chronic-somatic or mental illness), students with disability (students with a self-reported disability or a health-related impairment), students with children (who raise at least one [own] child under the age of 18), full-time employed students (students who are employed for at least 30 h per week), older students (students who are older than 30 years of age), younger students (students who are up to 30 years of age), students with migration background (students who have at least one parent who was born in another country or who were born in another country themselves), and students with non-German native language (students who have a native language other than German). When participants indicated that they did not identify with any of the 10 groups, the option to choose other responses as well was deactivated.

All further self-report measures were assessed on a five-point Likert scale from “does not apply at all” to “fully applies.” *Social identity threat* was measured separately for each group the students indicated with four items based on Martiny and Nikitin (2019): “I am concerned that I will confirm stereotypes about the abilities of #Group# at university,” “I am concerned that stereotypes about #Group# might hinder my performance at university,” “I am concerned that the stereotypes about #Group# are true,” “I am concerned that the stereotypes about #Group# might influence how others judge my performance at university.”

*Sense of belonging to university* was measured with an eight-item scale based on Good et al. (2012): “At the FernUniversität, I feel accepted,” “At the FernUniversität, I feel respected,” “At the FernUniversität, I feel valued,” “At the FernUniversität, I feel appreciated,” “I feel like I belong at the FernUniversität,” “I feel like a member of the group of students at the FernUniversität,” “I feel connected to other students at the FernUniversität,” “I feel like I am a part of the students at the FernUniversität.” The same eight items were applied for sense of belonging to the CSCL group by only replacing “university” by “study group.”

*Social approach motivation* was measured with four items based on Gable (2006) which were already applied in the context of higher education by Froehlich et al. (2022): “I try to get a deeper relationship with other students,” “I try to get relationships with other students that develop positively,” “I try to strengthen bonds and intimacy in my relations to other students,” “I try to share many fun and meaningful experiences with other students.”

*Behavioral intentions for contact* were assessed with three items based on Froehlich et al. (2022): “I plan to join an organization to meet other students in the near future,” “I will contact other students to start/join a study group in the near future,” and “I will volunteer at various events that the university holds in the near future.”

*Off-system behavior,* a control variable, was measured with three self-generated items: “In our group, we have used other media than Moodle for task-related exchange,” “In our group, we have used other media than Moodle for content-related collaboration on the task,” “In our group, we have used other media than Moodle for personal exchange.” Further measures collected for the superordinate project are available on request.

#### 2.2.2 Behavioral measures

During each phase of the collaboration (getting to know each other, CSCL phase 1, CSCL phase 2), forum activity in the course-related Moodle discussion boards was collected. Applying social network analysis, we computed *discussion outdegree* (i.e., number of replies to other students’ threads in the course-related Moodle forum) as our main behavioral measure for student interaction.

Access to the data on student activity in the Etherpad Lite was not available at the time of the pre-registration of the present study. We only received access to this measure after the end of the semester and included exploratory analyses with the Etherpad Lite data as a measure of actual task collaboration. With another social network analysis, we computed *Etherpad outdegree* (i.e., edits made by a student in a text that has been written by another student). Additionally, *Etherpad text edits* (i.e., sum of all operations of a student in the text, e.g., writing/ deleting/ copying/ pasting/ formatting) was investigated for the exploratory analyses since these measures best represented behavior aligned with social approach motivation in the CSCL context. Furthermore, key-strokes, clickstreams, and scroll data in the Etherpads were collected for analyses in the scope of another project.

### 2.3 Data analysis

All multi-item measures had sufficient reliability (*α*s > 0.82) and were aggregated into scales. Social identity threat was additionally aggregated across all groups to which each student had responded. For cross-sectional and longitudinal mediation analyses, all predictor variables were z-standardized. Because of the large differences in range and variances between self-report and behavioral data, we z-standardized all predictor and outcome variables before computing bivariate correlations. Since RI-CLPM consider only one level of nested data in the exploratory analyses (i.e., measurements nested within individuals), we accounted for the second level (i.e., students nested in CSCL groups) by conducting robustness checks including clustering for the CSCL group level for all pre-registered Hypotheses. Detailed results of robustness checks are reported in Supplementary material S1. We applied robust Maximum Likelihood estimation to consider the outcome variable of discussion outdegree which violated the assumption of normality as it was left-skewed. Additionally, we implemented Full-Information Maximum Likelihood estimation to take missing data into account.

The forum interaction data was represented as a social network graph *G* = (*S*, *L*), where S is a set of nodes representing forum participants *s* and *L* is a set of directed edges of which the included elements are called *links* representing forum posts of respondents answering forum posts of original posters. Social network centrality measures were computed to analyze interactions within Moodle forum discussions. Each response to a forum post was interpreted as establishing a directed *link l* {s_x_, s_y_} between two participants, where the respondent *s_x_* is connected to the original poster *s_y_*. *Discussion outdegree* was calculated as the number of outgoing links (answers to different original posters) from each student, reflecting their social connection in the forum discussions. This approach allowed for a comprehensive analysis of the social dynamics within the Moodle forums, identifying key actors and understanding the flow of interactions.

Etherpad Lite stores individual text edits of the collaborative text editor using the Easysync Protocol which encodes the affected characters, their position in the text, and the applied formatting operations. The edits collected from Etherpad Lite were used to determine the overall number of edits per students and time period. For each edit, the authors of the character on the left and right side of the inserted or removed character were identified at the respective time and document status. Thus, we counted how often a student had changed the document at a specific position in the text given that the neighboring characters were previously contributed by themselves or another student in the same group. Furthermore, it was counted whose text has been added to or deleted by another student. In this way, it was possible to quantify students’ task-related collaboration in the text. For social network analysis, a graph *G* = (*S*, *L*) was constructed, where *S* is a set of *nodes* representing students *s* in the group, and *L* is a set of paired and directed vertices of which the included elements are called *links*. Each *link l* {s_x_, s_y_} represents an editing operation of student *s_x_* directly next to the text that was formerly added by *s_y_*. We calculated *Etherpad outdegree* as the outdegree of a node which indicates the number of times a student *s* made changes (adding, removing, formatting) to a text that was previously contributed by another student in the same group. Although it is most closely aligned with self-reported social approach motivation and *discussion outdegree*, it reflects only a limited range of all potential collaborative writing activities. Therefore, we also computed *Etherpad text edits* to get a more encompassing measure of what each student contributed to the CSCL assignment.

Hypothesis testing was conducted with R version 4.2.2 and RStudio version 2023.06.0 + 421 (R Core Team, 2022; Posit team, 2023). To investigate Research Question 1, we computed an analysis of variance (ANOVA) with Bonferroni-corrected post-hoc tests with stats (R Core Team, 2022). Cross-sectional Hypotheses (H1 and H2) were tested with path analyses using lavaan (Rosseel, 2012). Longitudinal Hypotheses (H3 and H4) were tested with RI-CLPM (Hamaker et al., 2015) using lavaan (Rosseel, 2012).

## 3 Results

Descriptive statistics and bivariate correlations of all variables included in the analyses are presented in Table 1. Social identity threat was negatively associated with sense of belonging but not with social approach motivation. Sense of belonging was positively associated with social approach motivation and behavioral intentions for contact with peers. Social approach motivation was positively associated with behavioral intentions. Discussion outdegree was not associated with any other measure.

**Table 1:** Means, standard deviations, and correlations of all variables.

Variable	*M*	*SD*	Social identity threat T1	Social identity threat T2	Social identity threat T3	Sense of belonging T1	Sense of belonging T2	Sense of belonging T3	Social approach motivation T1	Social approach motivation T2	Social approach motivation T3	Behavioral intentions T1
Social identity threat T1	1.7	0.7										
Social identity threat T2	1.6	0.7	0.70** [0.66, 0.74]									
Social identity threat T3	1.7	0.8	0.69** [0.64, 0.73]	0.73** [0.69, 0.77]								
Sense of belonging T1	3.8	0.8	−0.21** [−0.27, −0.14]	−0.17** [−0.24, −0.09]	−0.19** [−0.27, −0.11]							
Sense of belonging T2	3.7	1.0	−0.13** [−0.21, −0.05]	−0.17** [−0.23, −0.11]	−0.19** [−0.26, −0.11]	0.47** [0.41, 0.53]						
Sense of belonging T3	3.7	1.0	−0.15** [−0.23, −0.07]	−0.12** [−0.19, −0.04]	−0.14** [−0.21, −0.07]	0.40** [0.33, 0.47]	0.68** [0.64, 0.72]					
Social approach motivation T1	2.8	1.0	0.03 [−0.04, 0.10]	0.03 [−0.05, 0.11]	0.06 [−0.03, 0.14]	0.32** [0.26, 0.38]	0.26** [0.19, 0.33]	0.18** [0.10, 0.26]				
Social approach motivation T2	2.5	1.1	0.00 [−0.08, 0.08]	0.02 [−0.05, 0.08]	−0.04 [−0.11, 0.04]	0.21** [0.14, 0.29]	0.33** [0.27, 0.38]	0.30** [0.27, 0.37]	0.69* [0.65, 0.73]			
Social approach motivation T3	2.5	1.1	−0.02 [−0.10, 0.06]	0.02 [−0.05, 0.10]	0.01 [−0.06, 0.08]	0.18** [0.10, 0.26]	0.26** [0.18, 0.33]	0.33** [0.26, 0.39]	0.67** [0.62, 0.71]	0.75** [0.71, 0.78]		
Behavioral intentions T1	2.5	0.9	0.05 [−0.02, 0.12]	0.02 [−0.06, 0.10]	0.08 [−0.00, 0.16]	0.22** [0.15, 0.29]	0.23** [0.15, 0.30]	0.21** [0.13, 0.29]	0.64** [0.59, 0.68]	0.54** [0.48, 0.60]	0.52** [0.45, 0.57]	
Behavioral intentions T2	2.4	1.0	0.03 [−0.05, 0.11]	0.09** [0.02, 0.15]	0.06 [−0.01, 0.14]	0.16** [0.08, 0.24]	0.23** [0.16, 0.29]	0.27** [0.20, 0.34]	0.60** [0.55, 0.65]	0.61** [0.57, 0.65]	0.60** [0.54, 0.64]	0.73** [0.69, 0.77]
Behavioral intentions T3	2.1	1.0	0.08 [−0.00, 0.16]	0.10** [0.03, 0.18]	0.12** [0.05, 0.19]	0.24** [0.16, 0.31]	0.20** [0.13, 0.28]	0.26** [0.20, 0.33]	0.48** [0.42, 0.54]	0.45** [0.39, 0.51]	0.58** [0.54, 0.63]	0.64** [0.59, 0.69]
Discussion outdegree T1	1.48	1.19	0.01 [−0.06, 0.09]	0.02 [−0.05, 0.09]	−0.00 [−0.08, 0.07]	−0.03 [−0.11, 0.04]	0.03 [−0.04, 0.10]	−0.03 [−0.11, 0.04]	−0.03 [−0.10, 0.05]	0.04 [−0.03, 0.11]	0.01 [−0.06, 0.08]	−0.08* [−0.15, −0.00]
Discussion outdegree T2	2.59	2.96	0.05 [−0.03, 0.13]	−0.00 [−0.08, 0.07]	−0.02 [−0.10, 0.07]	−0.10* [−0.18, −0.02]	−0.02 [−0.09, 0.06]	−0.01 [−0.09, 0.07]	−0.04 [−0.12, 0.04]	−0.06 [−0.13, 0.02]	−0.04 [−0.12, 0.04]	−0.06 [−0.14, 0.02]
Discussion outdegree T3	1.45	1.48	0.07 [−0.03, 0.17]	0.05 [−0.05, 0.14]	0.04 [−0.06, 0.14]	0.00 [−0.10, 0.10]	0.00 [−0.09, 0.10]	−0.06 [−0.16, 0.03]	−0.03 [−0.13, 0.07]	−0.03 [−0.12, 0.07]	−0.05 [−0.15, 0.05]	−0.12* [−0.22, −0.02]
Groupsize T1	4.4	1.4	0.01 [−0.06, 0.08]	0.06 [−0.02, 0.14]	0.04 [−0.04, 0.12]	0.02 [−0.05, 0.09]	0.04 [−0.04, 0.12]	0.04 [−0.05, 0.12]	−0.05 [−0.12, 0.02]	0.03 [−0.05, 0.11]	−0.01 [−0.10, 0.07]	−0.06 [−0.13, 0.01]
Groupsize T2	4.8	1.5	0.06 [−0.02, 0.14]	0.05 [−0.02, 0.11]	0.09* [0.01, 0.17]	−0.01 [−0.09, 0.07]	0.05 [−0.02, 0.11]	0.11** [0.03, 0.18]	−0.04 [−0.12, 0.04]	−0.01 [−0.07, 0.06]	0.00 [−0.07, 0.08]	−0.03 [−0.11, 0.05]
Groupsize T3	4.3	1.3	0.05 [−0.03, 0.13]	−0.00 [−0.08, 0.08]	0.04 [−0.02, 0.11]	0.09* [0.01, 0.18]	0.10** [0.03, 0.18]	0.08* [0.02, 0.15]	−0.02 [−0.10, 0.06]	0.02 [−0.05, 0.10]	0.03 [−0.04, 0.10]	−0.03 [−0.11, 0.05]
Off-system behavior T1	3.3	1.1	−0.03 [−0.10, 0.04]	−0.03 [−0.11, 0.05]	0.00 [−0.08, 0.08]	0.16** [0.09, 0.22]	0.06 [−0.02, 0.14]	−0.01 [−0.09, 0.07]	0.16** [0.09, 0.23]	0.11** [0.03, 0.18]	0.11** [0.03, 0.19]	0.12** [0.05, 0.18]
Off-system behavior T2	3.2	1.0	−0.03 [−0.11, 0.05]	−0.01 [−0.07, 0.06]	−0.01 [−0.08, 0.07]	0.15** [0.07, 0.23]	0.17** [0.10, 0.23]	0.12** [0.05, 0.20]	0.11** [0.03, 0.19]	0.14** [0.07, 0.20]	0.08 [−0.00, 0.15]	0.08 [−0.00, 0.15]
Off-system behavior T3	3.3	1.0	0.05 [−0.03, 0.13]	−0.07 [−0.15, 0.01]	0.00 [−0.06, 0.07]	0.12** [0.04, 0.20]	0.20** [0.13, 0.28]	0.18** [0.11, 0.24]	0.07 [−0.01, 0.16]	0.12** [0.04, 0.19]	0.07* [0.00, 0.14]	0.05 [−0.03, 0.14]
Etherpad outdegree T2	148.24	195.14	0.05 [−0.02, 0.12]	0.01 [−0.06, 0.07]	−0.00 [−0.07, 0.07]	−0.06 [−0.13, 0.01]	−0.06 [−0.13, 0.00]	−0.01 [−0.08, 0.06]	−0.03 [−0.10, 0.04]	0.02 [−0.05, 0.08]	0.00 [−0.07, 0.07]	−0.04 [−0.11, 0.03]
Etherpad outdegree T3	119.68	186.00	0.02 [−0.05, 0.09]	−0.02 [−0.09, 0.05]	−0.04 [−0.11, 0.03]	−0.01 [−0.08, 0.07]	−0.02 [−0.08, 0.05]	0.06 [−0.01, 0.13]	−0.02 [−0.09, 0.05]	0.05 [−0.01, 0.12]	0.06 [−0.01, 0.13]	−0.06 [−0.13, 0.01]
Etherpad text edits T2	936.2	1121.1	0.06 [−0.01, 0.13]	0.04 [−0.02, 0.11]	0.02 [−0.05, 0.09]	−0.09* [−0.16, −0.02]	−0.15** [−0.21, −0.08]	−0.12** [−0.19, −0.05]	−0.03 [−0.10, 0.04]	−0.02 [−0.09, 0.04]	−0.04 [−0.10, 0.03]	−0.06 [−0.13, 0.01]
Etherpad text edits T3	694.8	876.8	0.01 [−0.07, 0.08]	−0.02 [−0.09, 0.05]	−0.02 [−0.09, 0.06]	−0.01 [−0.09, 0.06]	−0.08* [−0.14, −0.01]	−0.04 [−0.11, 0.03]	−0.06 [−0.13, 0.01]	−0.01 [−0.08, 0.06]	−0.02 [−0.09, 0.05]	−0.11** [−0.18, −0.03]

Variable	Behavioral intentions T2	Behavioral intentions T3	Discussion outdegree T1	Discussion outdegree T2	Discussion outdegree T3	Groupsize T1	Groupsize T2	Groupsize T3	Off-system behavior T1	Off-system behavior T2
Behavioral intentions T3	0.71** [0.67, 0.74]									
Discussion outdegree T1	−0.08* [−0.15, −0.01]	−0.07 [−0.14, 0.01]								
Discussion outdegree T2	−0.09* [−0.16, −0.01]	−0.06 [−0.14, 0.02]	0.32** [0.25, 0.38]							
Discussion outdegree T3	−0.07 [−0.16, 0.02]	−0.02 [−0.12, 0.08]	0.41** [0.33, 0.48]	0.56** [0.50, 0.62]						
Groupsize T1	−0.02 [−0.10, 0.06]	−0.03 [−0.12, 0.05]	0.14** [0.07, 0.21]	0.01 [−0.07, 0.10]	0.00 [−0.10, 0.10]					
Groupsize T2	−0.01 [−0.08, 0.05]	−0.01 [−0.08, 0.07]	0.07 [−0.00, 0.14]	−0.03 [−0.10, 0.05]	−0.08 [−0.17, 0.01]	0.47** [0.41, 0.53]				
Groupsize T3	−0.01 [−0.09, 0.06]	0.00 [−0.07, 0.07]	0.08* [0.00, 0.15]	−0.07 [−0.15, 0.01]	−0.04 [−0.14, 0.05]	0.46** [0.39, 0.52]	0.48** [0.41, 0.53]			
Off-system behavior T1	0.08* [0.00, 0.16]	0.08 [−0.00, 0.16]	−0.16** [−0.23, −0.08]	−0.28** [−0.35, −0.20]	−0.20** [−0.29, −0.10]	0.03 [−0.04, 0.10]	0.07 [−0.01, 0.15]	0.06 [−0.02, 0.14]		
Off-system behavior T2	0.11** [0.04, 0.17]	0.05 [−0.02, 0.13]	−0.10** [−0.17, −0.03]	−0.39** [−0.46, −0.33]	−0.25** [−0.33, −0.16]	0.12** [0.04, 0.19]	0.06 [−0.00, 0.13]	0.15** [0.08, 0.23]	0.37** [0.30, 0.44]	
Off-system behavior T3	0.08 [−0.00, 0.15]	0.02 [−0.05, 0.09]	−0.12** [−0.19, −0.05]	−0.38** [−0.44, −0.31]	−0.30** [−0.39, −0.21]	0.08 [−0.01, 0.16]	0.07 [−0.00, 0.15]	0.13** [0.06, 0.19]	0.27** [0.20, 0.35]	0.74** [0.71, 0.78]
Etherpad outdegree T2	−0.02 [−0.08, 0.05]	−0.03 [−0.10, 0.04]	0.10** [0.04, 0.16]	0.18** [0.12, 0.25]	0.14** [0.06, 0.22]	0.01 [−0.06, 0.08]	0.01 [−0.06, 0.07]	0.06 [−0.01, 0.13]	−0.08* [−0.15, −0.01]	−0.08* [−0.14, −0.01]
Etherpad outdegree T3	0.02 [−0.05, 0.08]	−0.00 [−0.07, 0.07]	0.06 [−0.01, 0.12]	0.08* [0.01, 0.15]	0.12** [0.03, 0.20]	−0.03 [−0.10, 0.05]	0.02 [−0.05, 0.09]	0.04 [−0.03, 0.11]	−0.04 [−0.11, 0.03]	−0.01 [−0.08, 0.05]
Etherpad text edits T2	−0.04 [−0.11, 0.02]	−0.03 [−0.10, 0.04]	0.05 [−0.02, 0.11]	0.09* [0.02, 0.16]	0.06 [−0.02, 0.14]	−0.03 [−0.10, 0.04]	−0.03 [−0.09, 0.04]	−0.00 [−0.07, 0.07]	−0.03 [−0.10, 0.04]	−0.10** [−0.16, −0.03]
Etherpad text edits T3	−0.02 [−0.08, 0.05]	−0.02 [−0.09, 0.05]	0.03 [−0.04, 0.09]	0.10** [0.03, 0.17]	0.09* [0.00, 0.17]	−0.03 [−0.10, 0.04]	−0.05 [−0.11, 0.02]	−0.02 [−0.09, 0.05]	−0.04 [−0.11, 0.04]	−0.07* [−0.14, −0.01]

Variable	Off-system behavior T3	Etherpad outdegree T2	Etherpad outdegree T3	Etherpad text edits T2
Etherpad outdegree T2	−0.11**[−0.18, −0.04]			
Etherpad outdegree T3	0.00[−0.07, 0.07]	0.24**[0.18, 0.29]		
Etherpad text edits T2	−0.12**[−0.19, −0.05]	0.57**[0.53, 0.61]	0.23**[0.17, 0.29]	
Etherpad text edits T3	−0.08*[−0.15, −0.01]	0.26**[0.20, 0.31]	0.61**[0.57, 0.65]	0.47**[0.42, 0.51]

Confidence intervals are depicted in square brackets at the 95% level. * indicates *p* < 0.05. ** indicates *p* < 0.01.

We report the results for sense of belonging to the CSCL group as the main mediator throughout the manuscript. The results of additional analyses with sense of belonging to the university as an alternative mediator were similar to the results for sense of belonging to the CSCL group except for one path that is shown in Supplementary Table S2e. Detailed results with sense of belonging to the university as a mediator are reported in Supplementary material S2.

### 3.1 Levels of social identity threat in different student groups

**Figure 2:**
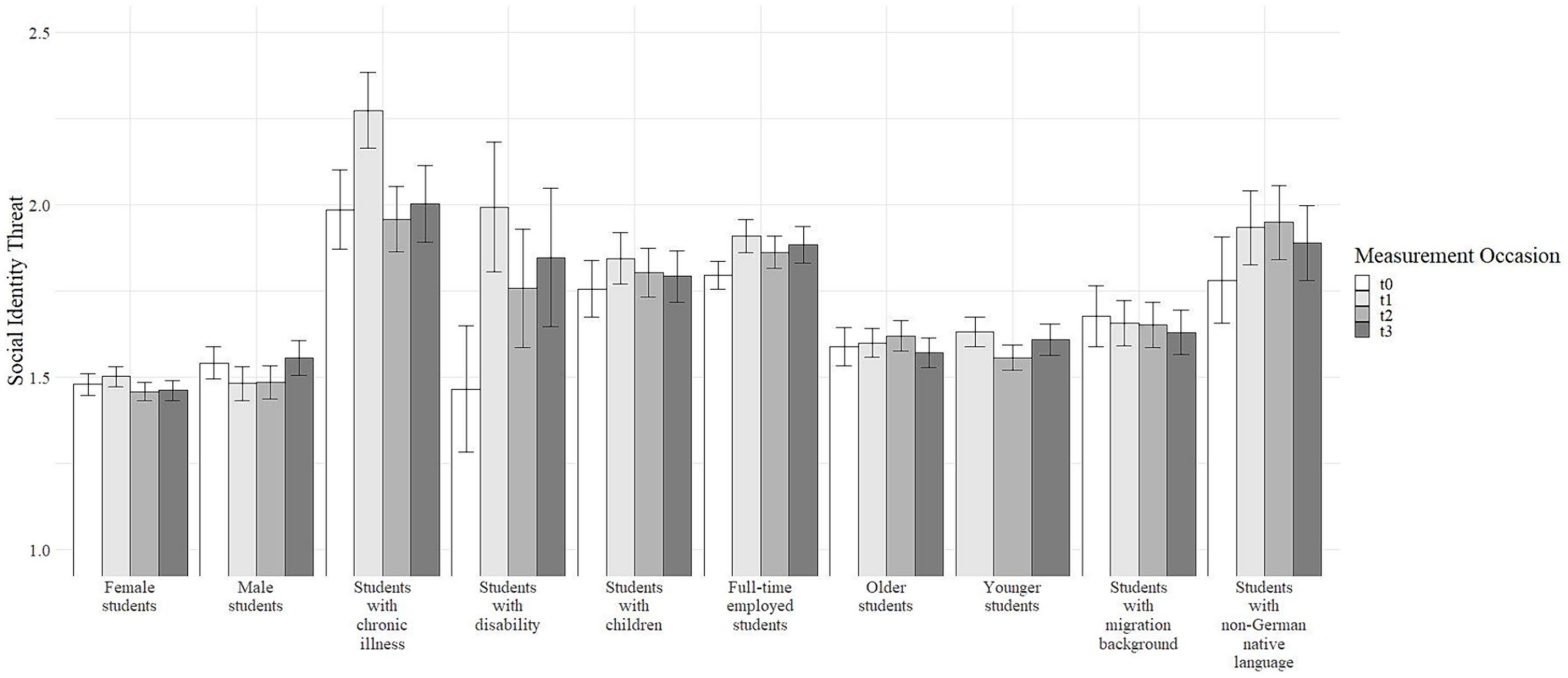
Means and standard errors of social identity threat for all student groups over all measurement occasions. Please note that values of social identity threat at T0 for younger students were not available due to a programming issue.

To investigate Research Question 1, we compared perceived social identity threat for different student groups at all measurement occasions. As depicted in Figure 2, students with chronic illness, students with disability, students with children, full-time employed students, and students with non-German native language reported higher levels of social identity threat across all measurement occasions, whereas female students, male students, older students, younger students, and students with migration background reported lower levels of social identity threat.

### 3.2 Cross-sectional analyses

**Figure 3:**
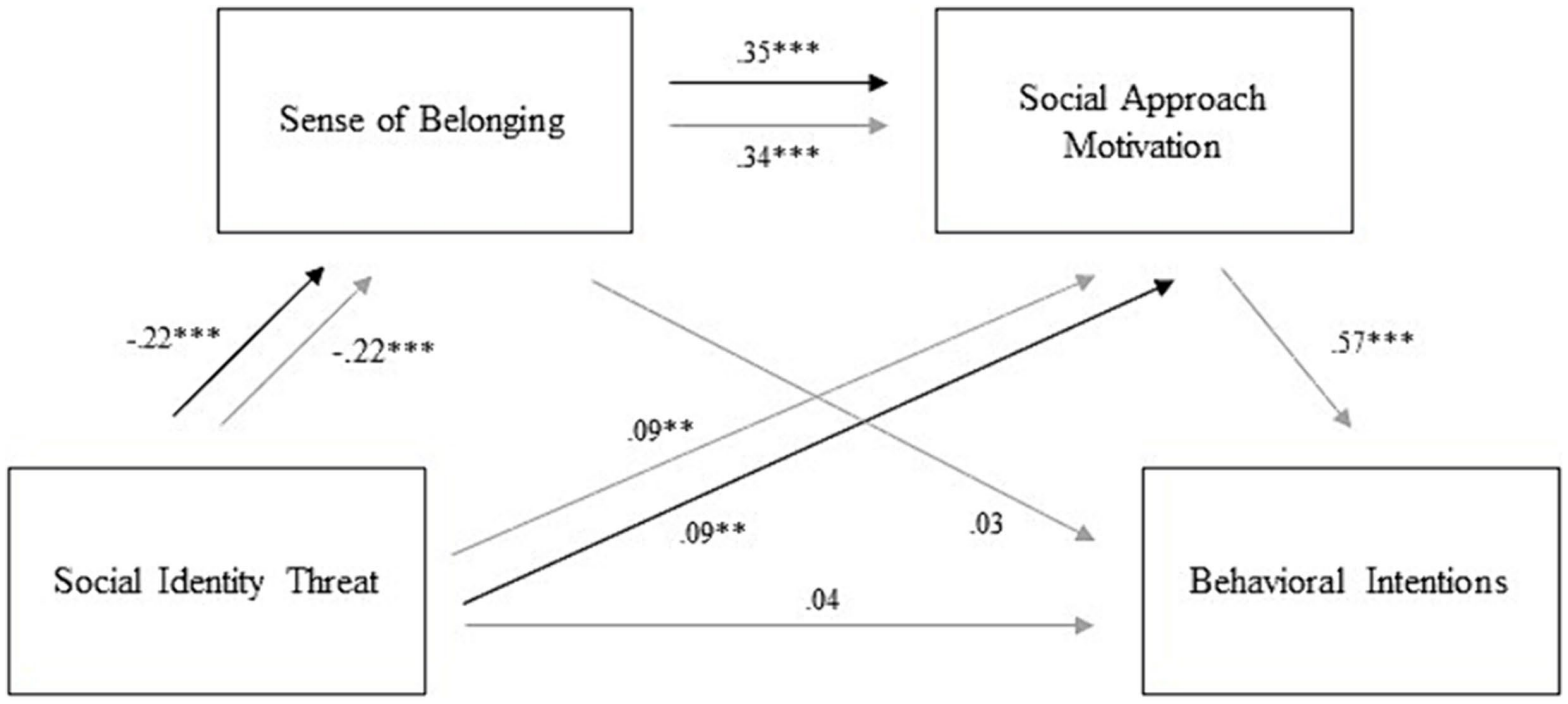
Path model for cross-sectional analyses. Black arrows depict paths and results for H1 whereas gray arrows depict paths and results for H2. * Indicates *p* < 0.05, ** indicates *p* < 0.01, and *** indicates *p* < 0.001.

Path models to test Hypotheses 1 and 2 are depicted in Figure 3. To investigate Hypothesis 1, we computed a manifest cross-sectional simple mediation model with social identity threat as the predictor, sense of belonging to the CSCL group as the mediator, and social approach motivation as the outcome. All variables were assessed at T1. The model was fully identified (i.e., included all possible paths). Results revealed that social identity threat negatively predicted sense of belonging, *β* = −0.22, 95% CI [−0.29, −0.15], *SE* = 0.03, *p* < 0.001, and positively predicted social approach motivation, *β* = 0.09, 95% CI [0.02, 0.16], *SE* = 0.03, *p* = 0.008. In turn, sense of belonging positively predicted social approach motivation, *β* = 0.35, 95% CI [0.28, 0.42], *SE* = 0.03, *p* < 0.001. As expected, the indirect effect was negative and significant, *β* = −0.08, 95% CI [−0.11, −0.05], *SE* = 0.01, *p* < 0.001, reflecting that social identity threat was associated with a reduced feeling of fitting in and in turn a reduced motivation to approach others.

To investigate Hypothesis 2, we computed a cross-sectional serial mediation model with social identity threat as the predictor, sense of belonging to the CSCL group as the first mediator, social approach motivation as the second mediator, and behavioral intentions for contact as the outcome. Again, the model was fully identified and all variables were assessed at T1. Results showed that social identity threat negatively predicted sense of belonging and positively predicted social approach motivation (see Table 2). Sense of belonging positively predicted social approach motivation. Social approach motivation positively predicted behavioral intentions for contact. Again, as expected the indirect effect was negative and significant. Results for cross-sectional Hypotheses did not differ in additional models including robustness checks (see Supplementary material).

**Table 2:** Results of the path model testing H2.

*Direct effects*												
		Consequent										
		Sense of belonging				Social approach motivation				Behavioral intentions		
Antecedent		*b* [95% CI]	*SE*	*p*		*b* [95% CI]	*SE*	*p*		*b* [95% CI]	*SE*	*p*
Social identity threat	a_1_	−0.22 [−0.29; −0.15]	0.03	<0.001	a_2_	0.09 [0.02; 0.16]	0.03	0.008	c_p_	0.04 [−0.01; 0.09]	0.03	0.043
Sense of belonging		–	–	–	d_21_	0.34 [0.28; 0.42]	0.03	<0.001	b_1_	0.03 [−0.02; 0.09]	0.03	0.268
Social approach motivation		–	–	–		–	–	–	b_2_	0.57 [0.52; 0.62]	0.03	<0.001

*Indirect effect*												
										*b* [95% CI]	*SE*	*p*
Social identity threat ➔ Social approach motivation ➔ Sense of belonging ➔ Behavioral intentions									a_1_d_21_b_2_	−0.04 [−0.06; −0.03]	0.01	<0.001

### 3.3 Longitudinal analyses

**Figure 4:**
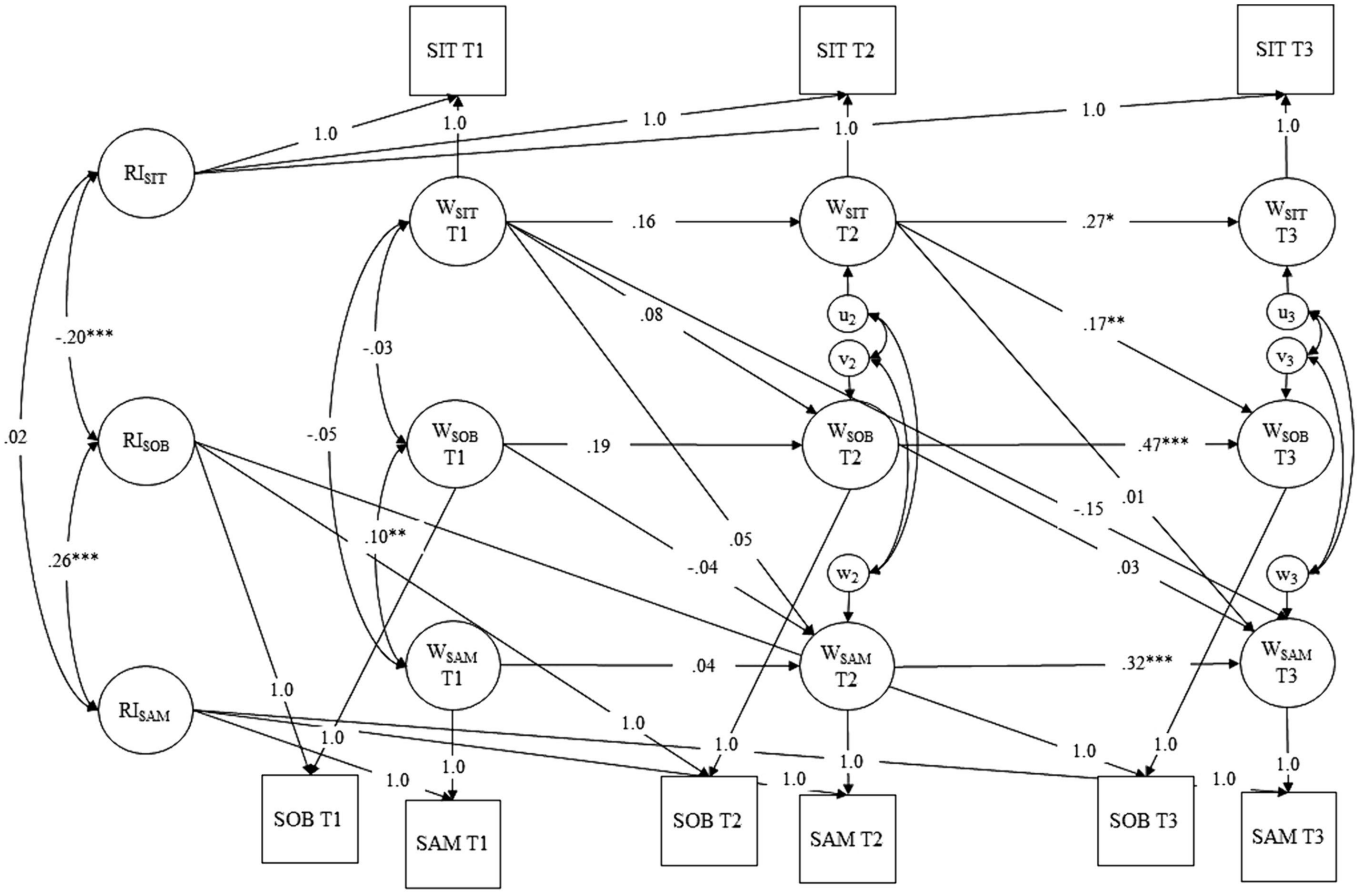
RI-CLPM for Hypothesis 3. Social identity threat is abbreviated with SIT, sense of belonging to the CSCL group is abbreviated wit SOB, and social approach motivation is abbreviated with SAM. Between-subject-variation is represented in RIs whereas within-subject variation is represented in Ws. * Indicates *p* < 0.05, ** indicates *p* < 0.01, and *** indicates *p* < 0.001.

To investigate Hypothesis 3, we conducted a RI-CLPM with social identity threat, sense of belonging to the CSCL group, and social approach motivation at the three measurement occasions (T1-T3). The models included autoregressive paths and the hypothesized within-person effects: social identity threat at earlier measurement occasions predicted sense of belonging to the CSCL group and social approach motivation at later occasions. Additionally, sense of belonging to the CSCL group at earlier occasions predicted social approach motivation at later occasions (see Figure 4). According to Hamaker et al. (2015) and our Hypotheses, we only allowed first-order autoregressive effects and cross-lagged paths from one measurement occasion to the next. The only path across two measurement occasions included in the model was the direct effect from social identity threat at T1 on social approach motivation at T3, since it was necessary for testing the longitudinal mediation Hypothesis. Random intercepts and residual variances were allowed to correlate. The model showed good fit, χ^2^ (8) = 11.73, RMSEA =0.02, CFI = 1.00, TLI = 0.99, SRMR = 0.01. Social identity threat at T2 positively predicted sense of belonging at T3, *β* = 0.17, *SE* = 0.06, *p* = 0.008. However, there were no further significant direct effects of social identity threat on sense of belonging and social approach motivation or of sense of belonging on social approach motivation (see Supplementary material S3). Furthermore, the expected within-person indirect effect of social identity threat at T1 on social approach motivation at T3 via sense of belonging to the CSCL group at T2 was not significant, *β* = 0.002, *SE* = 0.01, *p* = 0.651. Results were similar when taking the clustering for the CSCL group into account as an additional robustness check. Interestingly, the random intercept for social identity threat was negatively correlated with the random intercept for sense of belonging to the CSCL group, *r* = −0.20, *SE* = 0.03, *p* < 0.001, which in turn was positively correlated with the random intercept for social approach motivation, *r* = 0.26, *SE* = 0.04, *p* < 0.001. In other words, stable across measurement occasions, students with social identity threat above the sample average reported sense of belonging to the CSCL group below the average. In turn, students with sense of belonging below the average had lower-than-average motivation for contact with other students.

**Figure 5:**
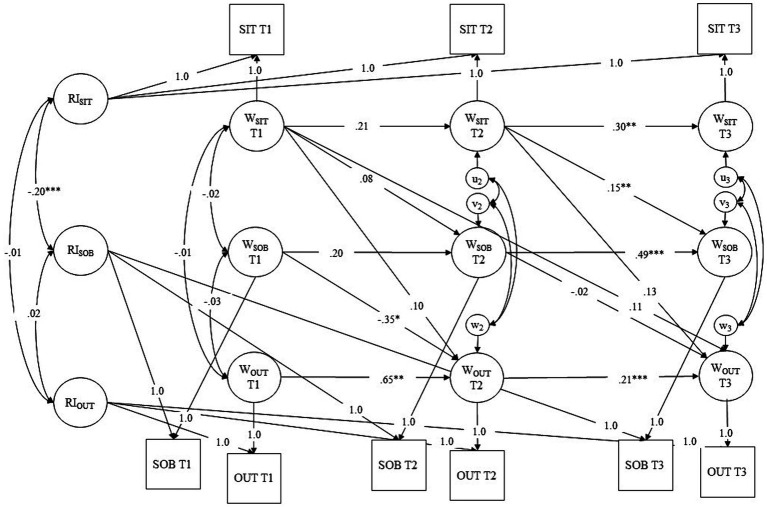
RI-CLPM for Hypothesis 4. Social identity threat is abbreviated with SIT, sense of belonging to the CSCL group is abbreviated wit SOB, and discussion outdegree is abbreviated with OUT. Between-subject-variation is represented in RIs whereas within-subject variation is represented in Ws. * Indicates *p* < 0.05, ** indicates *p* < 0.01, and *** indicates *p* < 0.001.

To investigate Hypothesis 4, we computed an analogous RI-CLPM with discussion outdegree as the behavioral outcome measure (see Figure 5). The RI-CLPM showed good fit, χ2 (8) = 5.82, RMSEA <0.001, CFI = 1.00, TLI = 1.01, SRMR = 0.01. Again, social identity threat at T2 positively predicted sense of belonging at T3, *β* = 0.15, *SE* = 0.06, *p* = 0.009. Sense of belonging at T1 negatively predicted outdegree at T2, *β* = −0.35, *SE* = 0.16, *p* = 0.030. No further significant direct effects from social identity threat on sense of belonging and discussion outdegree or from sense of belonging on discussion outdegree were found. In contrast to our expectations, there was no significant indirect effect of social identity threat on discussion outdegree via sense of belonging to the CSCL group on the within-person-level, *β* = −0.002, *SE* = 0.01, *p* = 0.802. Results were similar when taking the clustering for the CSCL group into account as an additional robustness check. On the between-person-level, the random intercept for social identity threat was negatively correlated with the random intercept for sense of belonging to the CSCL group, *r* = −0.20, *SE* = 0.03, *p* < 0.001. However, the random intercept for discussion outdegree was not significantly correlated with the random intercepts for social identity threat or sense of belonging to the CSCL group. Detailed results of H4 are reported in Supplementary material S4.

### 3.4 Exploratory analyses

Since Etherpad Lite data were available only for the two CSCL phases, RI-CLPM could not be computed. As analyses with the Etherpad Lite data were exploratory, we report bivariate correlations of social identity threat, sense of belonging, discussion outdegree, Etherpad outdegree, and Etherpad text edits (see Table 1). Similar to the outcomes of the main analyses, neither Etherpad outdegree nor Etherpad text edits were significantly related to social identity threat. Etherpad outdegree was also not significantly related to sense of belonging to the CSCL group. However, Etherpad text edits in working phase 1 were negatively related to sense of belonging to the CSCL group at all measurement occasions. Etherpad text edits in working phase 2 were negatively related to sense of belonging to the CSCL group at T2.

Since the number of active students in the CSCL groups might have influenced sense of belonging to the CSCL group and activity in the group (in the forum as well as in the Etherpads), we repeated all cross-sectional analyses controlling for group size. Results did not change when controlling for group size (see Supplementary material S5). In longitudinal analyses, the models did not converge when group size was added as control variable due to insufficient model identification. Correlations revealed that group size at T2 was positively related to social identity threat at T3, *r* = 0.09, 95% CI = [0.01, 0.17] and to sense of belonging to the CSCL group at T3, *r* = 0.11, 95% CI = [0.03, 0.18]. Group size at T3 was positively related to sense of belonging to the CSCL group at T1, *r* = 0.09, 95% CI = [0.01, 0.18], T2, *r* = 0.10, 95% CI = [0.03, 0.18], and T3, *r* = 0.08, 95% CI = [0.02, 0.15].

## 4 Discussion

This study contributes to understanding the social consequences of perceived social identity threat in distance learning when there is no face-to-face contact between students. First, we showed that perceived social identity threat varies between different student groups in higher distance education. Second, we replicated the cross-sectional findings from face-to-face contexts in higher distance education, namely, the mediation of social identity threat on social approach motivation via lower sense of belonging. Third, the current study has shown that within- and between-person effects need to be separated. Fourth, based on research showing an intention-behavior gap, we included self-reported and behavioral outcome measures collected via Learning Analytics in the same study.

### 4.1 (Some) non-traditional student groups are particularly at risk of experiencing social identity threat in CSCL

In line with research on stereotypes about non-traditional students in higher distance education (Bick et al., 2022), we found that social identity threat differed between student groups. After this earlier research has demonstrated that negative competence-related stereotypes about specific student groups are widespread in higher distance education, the present study goes beyond this by showing that negative stereotypes also threaten these students’ social identities. In detail, the non-traditional student groups (i.e., students with chronic illness, students with disability, students with children, full-time employed students, students with non-German native language) that we found to report higher levels of social identity threat show a strong overlap with the groups that were negatively stereotyped on ability in Bick et al. (2022) (i.e., students with migration background, students with chronic illness, students with disability, students with non-German native language). Interestingly, the traditional student groups that reported lower values of social identity threat in our study (i.e., male students, younger students) were the groups with the lowest values on the competence-related stereotype facets of conscientiousness (both) and ability (younger students only). However, these divergent findings can be explained by the fact that although male and younger students are negatively stereotyped in the specific context of a psychology course at a distance university where most students are female and older than 30 years, these groups do not often face negative competence-related stereotypes in other contexts (e.g., Fiske et al., 2002). Furthermore, since informal communication at a distance university is not common, these groups might not even be aware of these negative stereotypes, which is a prerequisite for social identity threat (Spencer et al., 2016). Thus, the present research substantiated earlier findings reporting higher vulnerability of non-traditional student groups in the context of higher distance education (e.g., Stoessel et al., 2015).

Female students and students with migration background reported comparably low levels of social identity threat in the current study, although both groups meet the definition of non-traditional students and were found to be negatively affected by social identity threat in face-to-face contexts in previous studies (Spencer et al., 1999; Froehlich et al., 2022). In the case of female students, the large proportion of female students in psychology (usually around 70%) might lead to lower social identity threat since females represent the majority group in this specific context. However, it remains an open question whether this result is generalizable or domain-specific. In contrast to the current research which investigated CSCL in the domain of scientific reading and writing, future research should investigate whether female students report higher social identity threat in psychology courses that reflect more traditional gender stereotypes about women’s mathematical competence (e.g., research methods and statistics; Martiny and Nikitin, 2019).

In the case of students with migration background, the following reasons might explain the comparably low levels of social identity threat. When asking which groups students identified with, both students with migration background and students with a non-German native language were presented. Only half of the students who identified as students with migration background also identified as having a non-German native language. Students who identified as students with migration background but perceive German to be their first language might be second- or third-generation immigrants and therefore might have developed dual social identities (i.e., simultaneously identifying with their ethnic group of origin and the national group of the residence country), which acts as a buffer against the social consequences of social identity threat (Froehlich et al., 2023). Students identifying with both student groups might have contrasted stereotypes about the two groups and attributed negative stereotypes and associated social identity threat to the group of students with non-German native language, resulting in lower levels of reported social identity threat regarding the identification with students with migration background. Future research is needed to verify this post-hoc explanation.

Interestingly, we found the highest levels of social identity threat among students with chronic illness. This result is surprising because based on the social identity model of deindividuation effects (SIDE; Postmes et al., 2001; Spears et al., 2002), we had expected social categories that are easily recognizable in online interactions such as gender, ethnicity/ native language, or age to be most salient in higher distance education. In contrast, it is often not immediately apparent in online contexts whether peers have a chronic illness or not. Nevertheless, students who identified as members of the group of students with chronic illness were relatively worried about being negatively stereotyped because of their group membership. In line with Clair et al. (2005), students with chronic illness may be repeatedly confronted with the need to disclose their chronic illness to their peers, as in collaborative contexts “those with an invisible chronic illness must find ways to explain or hide symptoms from others” (Clair et al., 2005, p. 91). Therefore, belonging to the group of students with chronic illness might be similarly relevant or, in line with our data, even more relevant than belonging to visible groups as social identity threat is intensified by the conflict of whether or not to disclose the chronic illness to peers.

The current research aggregated self-reported social identity threat across all students in the data set and did therefore not consider the extent to which students identified with one or another group. In the present study, sample sizes for subgroups of non-traditional students were too small to examine whether the associations between social identity threat and the outcome variables differed between the traditional and non-traditional student subgroups (see Supplementary material S6). Furthermore, students were clustered in CSCL groups, so that not only membership of individual students to sociodemographic groups was important, but also the multi-attributional diversity at the CSCL group level. Research at the group level has shown that in groups with high sociodemographic diversity, task-related prior knowledge and skills cannot be optimally utilized for group performance (Voltmer et al., 2022a,b, 2024). Future research should therefore simultaneously consider the membership of individual students in sociodemographic groups targeted by negative stereotypes and the impact of the demographic composition of the CSCL groups. For example, van Dijk et al. (2017) explain that stereotypes and social identity threat play an important role for microdynamics in teams that can impact collaboration and performance. It was however beyond the scope of the current manuscript to investigate these complex interrelations.

### 4.2 Self-reported consequences of social identity threat in CSCL

The present study aimed to replicate and better understand the social consequences of social identity threat shown in face-to-face contexts (Martiny and Nikitin, 2019; Rahn et al., 2021; Froehlich et al., 2023) in the emerging and important context of higher distance education. Since findings from face-to-face contexts cannot directly be translated to online collaborative learning contexts (Kreijns et al., 2024), we argue that testing the generalizability of earlier findings to higher distance education settings was a crucial part in this study. We replicated previous results on the mediating role of sense of belonging for the association between social identity threat, social approach motivation, and behavioral intentions for peer contact in cross-sectional analyses. This result indicates that findings on social identity threat from traditional educational contexts with face-to-face classrooms can be generalized to digital educational contexts with mostly asynchronous computer-mediated communication. These findings further support that group membership is especially salient in online contexts which amplifies the likelihood of stereotype activation (Postmes et al., 2002; Spears et al., 2002) and in turn, social identity threat and its consequences.

In contrast, the longitudinal investigation of social identity threat, sense of belonging, and social approach motivation revealed unexpected findings. Due to the repeated measurements implemented during a CSCL task over several weeks, we were able to conduct state-of-the-art analyses including RI-CLPM. As Hamaker et al. (2015) emphasize, the main advantage of the RI-CLPM is its ability to disentangle within- and between-persons components of variance in longitudinal data. Consequently, the application of the RI-CLPM allowed us to investigate whether the hypothesized mediation model reflects psychological intraindividual processes that unfold over time. The results of the current study do not support this. Interestingly, the correlations of the random intercepts, which represent a stable between-person variance, indicated that the mediation might be a between-person effect. Students with a higher average social identity threat over time also had lower average levels of sense of belonging to the CSCL group and in turn a lower average social approach motivation over time. Such an association of the random intercepts would argue for more stable rank-order effects in a given classroom or course, rather than psychological processes unfolding in individuals over time. This finding supports and specifies the mediation effect that has been found in previous research (Martiny and Nikitin, 2019; Froehlich et al., 2022, 2023).

### 4.3 Behavioral consequences of social identity threat in CSCL

In addition to self-reported social approach motivation and behavioral intentions, the current study used learning analytics to compute social network analysis based on data generated during the actual CSCL collaboration. The main behavioral outcome variable was discussion outdegree, reflecting a student’s responses to postings of their peers in course-related Moodle forums. Contrary to our expectations, discussion outdegree was unrelated to our main predictor variables of social identity threat and sense of belonging. One possible explanation for the diverging results for the self-report and the behavioral data is that there is a gap between intention and behavior as actual collaborative behavior might be influenced by additional factors beyond social approach motivation and behavioral intentions to interact with peers. For example, students might have wanted to interact more with peers but did not have time, e.g., due to family commitments. An alternative explanation is that discussion outdegree encompasses merely students’ discussion about the CSCL task, but not actual collaborative writing activities. Our data on this task-related discussion are probably also incomplete, as students might have used not only the designated Moodle forums, but also other communication channels outside of the learning environment. In fact, students indicated that they used other media than the course-related Moodle forum for communication about the course (i.e., off-system behavior). However, statistically controlling for off-system behavior did not change the results of cross-sectional analyses (see Supplementary material S7).

In exploratory analyses, we investigated correlations between social identity threat and sense of belonging with further behavioral outcomes collected via Etherpad Lite instances for each CSCL group. Etherpad outdegree and Etherpad text edits reflect the closest measures of actual collaborative behavior in the current study. Similar to the results for discussion outdegree, Etherpad outdegree was also unrelated to social identity threat and sense of belonging. It should be noted that Etherpad outdegree reflects only the edits a student made in the text written by another student, but no initial writing of the text or edits made in the text that was initially written by themselves. Furthermore, as the behavioral measure in Moodle (i.e., discussion outdegree), we only considered outdegree representing outgoing activity from a student but not indegree representing incoming activity like messages that were sent to this person or number of answers towards their threads in Moodle or changes another student had made in their text in the Etherpads, respectively. Since actual interaction and not only the initiation of contact is relevant for social outcomes like belonging (Walton and Cohen, 2007), a more comprehensive investigation of social network analysis measures might be reasonable for future studies. Etherpad text edits were unrelated to social identity threat but negatively related to sense of belonging to the CSCL group. Research about frustration in CSCL at a Spanish distance learning university revealed that the main source of frustration in CSCL was imbalance of commitment in group tasks which was further described as “sometimes I run into someone whose contribution was almost nothing. When that happens, I tend to do more than I can, to compensate, and this makes me feel nervous, causes some discomfort and feeling of injustice” (Capdeferro and Romero, 2012). In line with these findings, it could be argued that also in the present sample, students were frustrated about being forced to invest more because other students invested less, independent of the size of the CSCL groups. As Capdeferro and Romero (2012) recommend, instructions on effective collaboration and the communication of realistic expectations for the course might help online learners to overcome frustration and to enhance belonging to the CSCL group in collaborative courses at distance learning universities.

We conclude that our behavioral outcomes (i.e., discussion outdegree, Etherpad outdegree, and Etherpad text edits) did not reveal any expected findings although they were carefully selected and collected in different platforms which should have ruled out effects of, for instance, off-system behavior. Due to the novelty of using this kind of behavioral data as proxy for task-related integration in a group, we recommend further research to focus on understanding why the behavior did not reflect the indicated intentions. With fine-grained collaboration data as it is provided in the Etherpads and with the large samples that can be collected in higher distance education institutions, much more detailed analyses can potentially reveal reasons for our findings. Further quantitative approaches can shed light on roles of specific students during a collaborative task (e.g., produce text, modify wording/ structure), temporal dimensions of collaboration (a−/synchronous) which could, maybe in combination with a qualitative investigation, complement the reasons for our findings.

### 4.4 Limitations and practical implications

Notwithstanding the outlined contributions of this work, some limitations should be discussed more extensively. First, the longitudinal analyses were conducted over a total of 8 weeks with intervals of 2–3 weeks, which might not have been enough time for the intraindividual effects to unfold (Cook et al., 2012). In addition, longitudinal studies with the goal of investigating cross-lagged associations should ideally be conducted under stable conditions. In contrast, in our study, the changing CSCL tasks in the different phases (getting to know each other, working phases 1 and 2) and the transition to a new academic institution (measurement during the first weeks of the first semester in the Bachelor’s program) were a source of high variability. This lack of stability over time is also reflected in the often weak or insignificant autoregressive paths in the longitudinal analyses. Future research should therefore replicate the results under more stable conditions in order to ensure more reliable interpretation of the reported results. Second, we cannot ensure generalizability of all our findings to other (distance) universities. We assume that the findings we replicated are well generalizable to other universities (i.e., non-traditional student groups reporting higher social identity threat, cross-sectional (serial) mediation of social consequences of social identity threat). However, due to the novelty of the longitudinal analyses, we encourage researchers to investigate comparable hypotheses at other universities to get more insights into whether our findings were specific to our design and sample. Third, as we aimed to understand the social consequences of social identity threat in a field study, only psychology students participated which clearly biased our sample regarding, e.g., gender. Furthermore, the voluntary participation in the study has probably led to selection effects (e.g., Wagner, 2012). However, we still collected a large sample size and found substantial variation in the data so we assume that our results adequately represent psychology students at a large distance university. Fourth, it was possible for participants to indicate self-identification with several student groups simultaneously. Due to the complex data structure (measurements nested in individuals nested in CSCL groups), we aggregated items of social identity threat across groups per individual. Adjusting for student groups as a further level of clustering in combination with the level of CSCL groups was not possible with current statistical software. An investigation of intersectionality (i.e., the psychological relevance of specific combinations of group memberships) was beyond the scope of the current study. Future research could investigate the extent and consequences of social identity threat for different intersections of student groups.

Despite these limitations, the present study has a high practical value for different stakeholders, especially in higher distance education. The finding that specific student groups are at risk of perceiving social identity threat implies that higher distance education institutions should develop and implement interventions to reduce social identity threat in these specific student groups to foster educational equity (Walton et al., 2015; Liu et al., 2021). Teachers in such institutions should be sensitized to the existence of negative effects of social identity threat in order to prevent them from even unintentionally activating stereotypes (e.g., by using biased study materials or language). Finally, students should be provided with information on potential issues arising from social identity threat and how to deal with it (Alter et al., 2010). The exploratory finding that the association of social identity threat, sense of belonging, and social approach motivation was a between- instead of the expected within-person effect indicates that for effectively reducing social identity threat, individuals prone to its negative consequences must be identified and specifically addressed which again underlines the above-mentioned need for tailored interventions. Since easily available information like sociodemographic group membership that has traditionally been used to determine whether a recipient will benefit from an intervention or not (e.g., Walton and Cohen, 2011) will most probably not adequately represent the individual students’ need for an intervention (but also, e.g., the setting; see Spencer et al., 2016), further research will be needed to identify individuals prone to social identity threat and its consequences based on reliable individual and situational characteristics. Using learning analytics for understanding and predicting students’ need for interventions is a promising route for further research with the potential to unfold more specific implications for higher distance education.

## 5 Conclusion

“To close achievement gaps, it is necessary both to eradicate psychological threats embedded in academic environments and to remove other barriers to achievement including objective biases, the effects of poverty, and so forth” (Walton and Spencer, 2009, p. 1137). The present study is a first step towards eliminating psychological threats in online learning environments, as it found that most non-traditional student groups in higher distance education are at risk of experiencing social identity threat. The present study thus underpins earlier research that points to problems faced by non-traditional student groups at university (e.g., Yildirim et al., 2021). Furthermore, we substantiated the mediation of social identity threat on social approach motivation via reduced sense of belonging by replicating it in the novel context of higher distance education. Finally, this study has taken a first step towards integrating learning analytics into research on social identity threat and belongingness in CSCL and found interesting effects that can be subject of future research aimed at improving the prerequisites of successful CSCL.

## Data availability statement

The datasets presented in this study can be found in online repositories. The names of the repository/repositories and accession number(s) can be found at: https://osf.io/axq7k/.

## Ethics statement

The studies involving humans were approved by Ethikkommission der Fakultät für Psychologie, FernUniversität in Hagen. The studies were conducted in accordance with the local legislation and institutional requirements. The participants provided their written informed consent to participate in this study.

## Author contributions

NB: Conceptualization, Formal analysis, Methodology, Visualization, Writing – original draft, Writing – review & editing, Investigation, Validation. LF: Conceptualization, Methodology, Supervision, Writing – review & editing, Investigation, Validation, Formal analysis, Resources. J-BV: Formal analysis, Methodology, Writing – review & editing, Data curation, Project administration, Conceptualization. JR: Project administration, Writing – review & editing, Investigation, Conceptualization. NR-S: Project administration, Writing – review & editing, Conceptualization. NS: Formal analysis, Resources, Writing – review & editing, Data curation, Conceptualization, Software. MB: Formal analysis, Resources, Software, Writing – review & editing, Conceptualization. SM: Writing – review & editing, Conceptualization, Resources. JN: Writing – review & editing, Conceptualization, Resources. SS: Project administration, Resources, Writing – review & editing, Conceptualization. AM: Methodology, Writing – review & editing, Conceptualization.

## Footnotes

^1^ https://osf.io/rymzb/

^2^ https://www.unipark.com/

^3^ https://github.com/ether/etherpad-lite

^4^ https://etherpad.org/
