# Supplementary material for: Virtually isolated: social identity threat predicts social approach motivation via sense of belonging in computer-supported collaborative learning
Source: Front Psychol. 2024 Sep 13;15:1346503. doi: 10.3389/fpsyg.2024.1346503 (PMC11427319; doi:10.3389/fpsyg.2024.1346503)
Supplement: Supplementary file 1 [file Data_Sheet_1.PDF]

Supplementary Material

Contents

Contents 1
1 Supplement 1: Robustness analyses clustering for CSCL group level 2
2 Supplement 2: Results of analyses with sense of belonging to university as mediator 10
3 Supplement 3: Results of analyses regarding H3 16
4 Supplement 4: Results of analyses regarding H4 17
5 Supplement 5: Results of analyses controlling for CSCL group size 19
6 Supplement 6: Frequencies of group identification per measurement occasion 21
7 Supplement 7: Results of analyses controlling for off-system behavior 22

1 Supplement 1: Robustness analyses clustering for CSCL group level

Table S1a

Results H1 with sense of belonging to group clustering for CSCL group level

		Direct Effects						
		Consequent						
		Sense of Belonging				Social Approach Motivation		
Antecedent		*b* [95% CI]	*SE*	*p*		*b* [95% CI]	*SE*	*p*
Social Identity Threat	a	-.22 [-.29; -.15]	.04	< .001	c	.09 [.01; .17]	.04	.022
Sense of Belonging		-	-	-	b	.35 [.27; .43]	.04	< .001
			Indirect Effect					
						b [95% CI]	*SE*	*p*
Social Identity Threat → Sense of Belonging → Social Approach Motivation					a*b	-.08 [-.11; -.06]	.02	< .001

Table S1b

Results H1 with sense of belonging to university clustering for CSCL group level

		Direct Effects						
		Consequent						
		Sense of Belonging				Social Approach Motivation		
Antecedent		*b* [95% CI]	*SE*	*p*		*b* [95% CI]	*SE*	*p*
Social Identity Threat	a	-.22 [-.29; -.15]	.04	< .001	c	.09 [.02; .17]	.04	.018
Sense of Belonging		-	-	-	b	.36 [.29; .43]	.04	< .001
			Indirect Effect					
						b [95% CI]	*SE*	*p*
Social Identity Threat → Sense of Belonging → Social Approach Motivation					a*b	-.08 [-.11; -.05]	. 02	< .001

Table S1c

Results H2 with sense of belonging to CSCL group clustering for CSCL group level

		Direct Effects										
		Consequent										
		Sense of Belonging				Social Approach Motivation				Behavioral Intentions		
Antecedent		*b* [95% CI]	*SE*	*p*		*b* [95% CI]	*SE*	*p*		*b* [95% CI]	*SE*	*p*
Social Identity Threat	a_1	-.22 [-.29; -.15]	.04	< .001	a_2	.09 [.02; .16]	.04	.022	c_p	.04 [-.001; .10]	.03	.111
Sense of Belonging		-	-	-	d_{21}	.34 [.27; .42]	.04	< .001	b_1	.03 [-.03; .09]	.03	.285
Social Approach Motivation		-	-	-		-	-	-	b_2	.57 [.52; .63]	.03	< .001
		Indirect Effect										
										b [95%CI]	*SE*	*p*
Social Identity Threat → Social Approach Motivation → Sense of Belonging → Behavioral Intentions									a_1*d_{12}*b_2	-.04 [-.06; -.03]	.01	< .001

Table S1d

Results H2 with sense of belonging to university clustering for CSCL group level

		Direct Effects										
		Consequent										
		Sense of Belonging				Social Approach Motivation				Behavioral Intentions		
Antecedent		*b* [95% CI]	*SE*	*p*		*b* [95% CI]	*SE*	*p*		*b* [95% CI]	*SE*	*p*
Social Identity Threat	a_1	-.22 [-.29; -.15]	.04	< .001	a_2	.09 [.02; .17]	.04	.018	c_p	.06 [-.001; .11]	.03	.028
Sense of Belonging		-	-	-	d_{21}	.35 [.28; .43]	.04	< .001	b_1	.01 [.04; .16]	.03	.001
Social Approach Motivation		-	-	-		-	-	-	b_2	.55 [.49; .61]	.03	< .001
		Indirect Effect										
										b [95%CI]	*SE*	*p*
Social Identity Threat → Social Approach Motivation → Sense of Belonging → Behavioral Intentions									a_1*d_{12}*b_2	-.04 [-.06; -.03]	.01	< .001

Table S1e

Results H3 with sense of belonging to CSCL group clustering for CSCL group level

	Unstardardized Beta	*SE*	*p*	Standardized Beta
Between-person effects				
Correlations				
Social Identity Threat → Sense of Belonging	-.20	.03	< .001	-.40
Social Identity Threat → Social Approach Motivation	.02	.03	.480	.03
Sense of Belonging → Social Approach Motivation	.26	.03	< .001	.49
Within-person effects				
Auto-regressive paths				
Social Identity Threat T2 → Social Identity Threat T1	.16	.15	.289	.15
Sense of Belonging T2 → Sense of Belonging T1	.19	.14	.176	.19
Social Approach Motivation T2 → Social Approach Motivation T1	.04	.17	.820	.03
Social Identity Threat T3 → Social Identity Threat T2	.27	.11	.015	.28
Sense of Belonging T3 → Sense of Belonging T2	.47	.09	< .001	.48
Social Approach Motivation T3 → Social Approach Motivation T2	.32	.08	< .001	.31
Cross-lagged paths				
Sense of Belonging T2 → Social Identity Threat T1	.08	.11	.463	.06

Social Approach Motivation T2 → Social Identity Threat T1	.05	.11	.615	.05
Social Approach Motivation T2 → Sense of Belonging T1	-.04	.10	.565	-.05
Sense of Belonging T3 → Social Identity Threat T2	.17	.06	.008	.13
Social Approach Motivation T3 → Social Identity Threat T2	.01	.07	.874	.01
Social Approach Motivation T3 → Sense of Belonging T2	.03	.05	.593	.03
Social Approach Motivation T3 → Social Identity Threat T1	-.15	.10	.135	-.13

Table S1f:

Results H3 with sense of belonging to university clustering for CSCL group level

	Unstardardized Beta	*SE*	*p*	Standardized Beta
Between-person effects				
Correlations				
Social Identity Threat → Sense of Belonging	-.21	.03	< .001	-.29
Social Identity Threat → Social Approach Motivation	.03	.03	.408	.04
Sense of Belonging → Social Approach Motivation	.33	.03	< .001	.44
Within-person effects				
Auto-regressive paths				
Social Identity Threat T2 → Social Identity Threat T1	.17	.17	.315	.15

Sense of Belonging T2 → Sense of Belonging T1	.04	.12	.736	.04
Social Approach Motivation T2 → Social Approach Motivation T1	.07	.17	.658	.06
Social Identity Threat T3 → Social Identity Threat T2	.28	.11	.010	.30
Sense of Belonging T3 → Sense of Belonging T2	.26	.11	.018	.25
Social Approach Motivation T3 → Social Approach Motivation T2	.37	.07	< .001	.36
Cross-lagged paths				
Sense of Belonging T2 → Social Identity Threat T1	.18	.10	.063	.20
Social Approach Motivation T2 → Social Identity Threat T1	.04	.11	.700	.04
Social Approach Motivation T2 → Sense of Belonging T1	.04	.11	.724	.03
Sense of Belonging T3 → Social Identity Threat T2	.07	.06	.233	.09
Social Approach Motivation T3 → Social Identity Threat T2	-.004	.07	.946	-.004
Social Approach Motivation T3 → Sense of Belonging T2	-.13	.10	.189	-.10
Social Approach Motivation T3 → Social Identity Threat T1	-.10	.10	.290	-.09

Table S1g:

Results H4 with sense of belonging to CSCL group clustering for CSCL group level

	Unstardardized Beta	*SE*	*p*	Standardized Beta
Between-person effects				
Correlations				
Social Identity Threat → Sense of Belonging	-.20	.03	< .001	-.41
Social Identity Threat → Discussion Outdegree	-.01	.05	.902	-.01
Sense of Belonging → Discussion Outdegree	.02	.04	.657	.05
Within-person effects				
Auto-regressive paths				
Social Identity Threat T2 → Social Identity Threat T1	.21	.16	.179	.19
Sense of Belonging T2 → Sense of Belonging T1	.20	.14	.156	.20
Social Approach Motivation T2 → Discussion Outdegree T1	.65	.22	.004	.22
Social Identity Threat T3 → Social Identity Threat T2	.30	.11	.005	.32
Sense of Belonging T3 → Sense of Belonging T2	.49	.09	< .001	.49
Discussion Outdegree T3 → Social Approach Motivation T2	.21	.06	< .001	.47
Cross-lagged paths				
Sense of Belonging T2 → Social Identity Threat T1	.08	.12	.463	.06
Discussion Outdegree T2 → Social Identity Threat T1	.10	.23	.667	.02
Discussion Outdegree T2	-.35	.15	.022	-.10

→ Sense of Belonging T1				
Sense of Belonging T3 → Social Identity Threat T2	.15	.06	.010	.12
Discussion Outdegree T3 → Social Identity Threat T2	.13	.12	.299	.06
Discussion Outdegree T3 → Sense of Belonging T2	-.02	.09	.805	-.01
Discussion Outdegree T3 → Social Identity Threat T1	.11	.16	.497	.05

Table S1h:

Results H4 with sense of belonging to university clustering for CSCL group level

	Unstardardized Beta	*SE*	*p*	Standardized Beta
Between-person effects				
Correlations				
Social Identity Threat → Sense of Belonging	-.21	.03	< .001	-.30
Social Identity Threat → Discussion Outdegree	-.01	.05	.810	-.02
Sense of Belonging → Discussion Outdegree	-.01	.03	.835	-.01
Within-person effects				
Auto-regressive paths				
Social Identity Threat T2 → Social Identity Threat T1	.21	.18	.236	.19
Sense of Belonging T2 → Sense of Belonging T1	.05	.13	.676	.06
Discussion Outdegree T2	.66	.23	.004	.22

→ Social Approach Motivation T1				
Social Identity Threat T3 → Social Identity Threat T2	.31	.11	.005	.33
Sense of Belonging T3 → Sense of Belonging T2	.27	.11	.017	.26
Discussion Outdegree T3 → Discussion OutdegreeT2	.20	.06	< .001	.46
Cross-lagged paths				
Sense of Belonging T2 → Social Identity Threat T1	.18	.10	.072	.20
Discussion Outdegree T2 → Social Identity Threat T1	.16	.23	.478	.03
Discussion Outdegree T2 → Sense of Belonging T1	.01	.25	.968	.002
Sense of Belonging T3 → Social Identity Threat T2	.07	.06	.222	.09
Discussion Outdegree T3 → Social Identity Threat T2	.11	.11	.344	.06
Discussion Outdegree T3 → Sense of Belonging T2	.09	.14	.552	.04
Discussion Outdegree T3 → Social Identity Threat T1	.09	.17	.602	.04

2 Supplement 2: Results of analyses with sense of belonging to university as mediator

Table S2a

Means and standard deviations of all variables and correlations of all variables with sense of belonging to university

Variable	*M*	*SD*	Sense of Belonging T1	Sense of Belonging T2	Sense of Belonging T3
Sense of Belonging T1	3.8	0.8			
Sense of Belonging T2	3.7	3.7	.72**<br>[.68, .76]		
Sense of Belonging T3	3.7	1.0	.72**<br>[.67, .75]	.80**<br>[.77, .82]	
Social Identity Threat T1	1.7	0.7	-.21**<br>[-.27, -.14]	-.13**<br>[-.21, -.05]	-.15**<br>[-.23, -.07]
Social Identity Threat T2	1.6	0.7	-.17**<br>[-.24, -.09]	-.17**<br>[-.23, -.11]	-.12**<br>[-.19, -.04]
Social Identity Threat T3	1.7	0.8	-.19**<br>[-.27, -.11]	-.19**<br>[-.26, -.11]	-.14**<br>[-.21, -.07]
Social Approach Motivation T1	2.8	1.0	.34**<br>[.28, .40]	.31**<br>[.24, .38]	.26**<br>[.19, .34]
Social Approach Motivation T2	2.5	1.1	.30**<br>[.23, .37]	.34**<br>[.28, .39]	.30**<br>[.23, .37]
Social Approach Motivation T3	2.5	1.1	.28**<br>[.20, .35]	.26**<br>[.19, .33]	.34**<br>[.28, .40]
Behavioral Intentions T1	2.5	0.9	.28**<br>[.22, .34]	.31**<br>[.23, .38]	.20**<br>[.12, .28]
Behavioral Intentions T2	2.4	1.0	.28**<br>[.22, .34]	.27**<br>[.20, .34]	.60**<br>[.55, .65]
Behavioral Intentions T3	2.1	1.0	.24**<br>[.16, .31]	.20**<br>[.13, .28]	.26**<br>[.20, .33]

Discussion Outdegree T1	1.48	1.19	-.03<br>[-.11, .04]	-.01<br>[-.08, .06]	-.04<br>[-.11, .04]
Discussion Outdegree T2	2.59	2.96	.00<br>[-.08, .08]	.02<br>[-.06, .10]	.03<br>[-.05, .11]
Discussion Outdegree T3	1.45	1.48	-.01<br>[-.12, .09]	.02<br>[-.07, .11]	.02<br>[-.07, .12]
Groupsize T1	4.4	1.4	-.04<br>[-.11, .03]	-.02<br>[-.10, .06]	.00<br>[-.08, .09]
Groupsize T2	4.8	1.5	-.03<br>[-.11, .05]	.04<br>[-.02, .11]	.04<br>[-.03, .12]
Groupsize T3	4.3	1.3	-.01<br>[-.09, .08]	.03<br>[-.05, .10]	.03<br>[-.04, .10]
Off-System Behavior T1	3.3	1.1	-.01<br>[-.08, .06]	.03<br>[-.05, .11]	-.07<br>[-.15, .01]
Off-System Behavior T2	3.2	1.0	-.01<br>[-.09, .07]	.05<br>[-.01, .12]	.04<br>[-.03, .12]
Off-System Behavior T3	3.3	1.0	-.02<br>[-.10, .06]	.08*<br>[.00, .16]	.05<br>[-.02, .12]
Etherpad Outdegree T2	148.24	195.14	-.04<br>[-.11, .03]	-.03<br>[-.09, .04]	.03<br>[-.04, .10]
Etherpad Outdegree T3	119.68	186.00	-.01<br>[-.08, .07]	-.02<br>[-.08, .05]	.06<br>[-.01, .13]
Etherpad Text Edits T2	936.2	1121.1	-.09*<br>[-.16, -.02]	-.15**<br>[-.21, -.08]	-.12**<br>[-.19, -.05]
Etherpad Text Edits T3	694.8	876.8	-.01<br>[-.09, .06]	-.08*<br>[-.14, -.01]	-.04<br>[-.11, .03]

Note. Confidence intervals are depicted in square brackets at the 95% level. * indicates $p < .05$. ** indicates $p < .01$.

Table S2b

Results H1 with sense of belonging to university

Antecedent		*Direct Effects* Consequent: Sense of Belonging *b* [95% CI]	*SE*	*p*		Social Approach Motivation *b* [95% CI]	*SE*	*p*
Social Identity Threat	a	-.22 [-.29; -.15]	.03	< .001	c	.09 [.03; .16]	.03	.007
Sense of Belonging		-	-	-	b	.36 [.29; .43]	.03	< .001
Indirect Effect						*b* [95% CI]	*SE*	*p*
Social Identity Threat → Sense of Belonging → Social Approach Motivation					a*b	-.08 [-.11; -.05]	.01	< .001

Table S2c

Results H2 with sense of belonging to university

Antecedent		*Direct Effects* Consequent: Sense of Belonging *b* [95% CI]	*SE*	*p*		Social Approach Motivation *b* [95% CI]	*SE*	*p*		Behavioral Intentions *b* [95% CI]	*SE*	*p*
Social Identity Threat	a_1	-.22 [-.29; -.15]	.03	< .001	a_2	.09 [.03; .15]	.03	.007	c_p	.06 [.01; .11]	.03	< .001
Sense of Belonging		-	-	-	d_{21}	.35 [.29; .42]	.03	< .001	b_1	.10 [.05; .15]	.03	< .001
Social Approach Motivation		-	-	-		-	-	-	b_2	.55 [.49; .60]	.03	< .001
Indirect Effects										*b* [95% CI]	*SE*	*p*
Social Identity Threat → Social Approach Motivation → Sense of Belonging → Behavioral Intentions									$a_1*d_{12}*b_2$	-.04 [-.06; -.03]	.01	< .001

Table S2d

Results H3 with sense of belonging to university

	Unstardardized Beta	*SE*	*p*	Standardized Beta
Between-person effects				
Correlations				
Social Identity Threat → Sense of Belonging	-.21	.03	<.001	-.40
Social Identity Threat → Social Approach Motivation	.02	.03	.480	.03
Sense of Belonging → Social Approach Motivation	.26	.03	<.001	.49
Within-person effects				
Auto-regressive paths				
Social Identity Threat T2 → Social Identity Threat T1	.17	.17	.317	.15
Sense of Belonging T2 → Sense of Belonging T1	.04	.14	.767	.04
Social Approach Motivation T2 → Social Approach Motivation T1	.07	.17	.656	.06
Social Identity Threat T3 → Social Identity Threat T2	.28	.11	.009	.30
Sense of Belonging T3 → Sense of Belonging T2	.26	.11	.016	.25
Social Approach Motivation T3 → Social Approach Motivation T2	.37	.071	<.001	.36
Cross-lagged paths				
Sense of Belonging T2 → Social Identity Threat T1	.18	.10	.056	.20

Social Approach Motivation T2 → Social Identity Threat T1	.04	.11	.700	.04
Social Approach Motivation T2 → Sense of Belonging T1	.04	.11	.718	.03
Sense of Belonging T3 →Social Identity Threat T2	.07	.07	.277	.09
Social Approach Motivation T3 → Social Identity Threat T2	-.004	.07	.948	-.004
Social Approach Motivation T3 → Sense of Belonging T2	-.13	.10	.178	-.10
Social Approach Motivation T3 → Social Identity Threat T1	-.10	.11	.331	-.09

Table S2e

Results H4 with sense of belonging to university

	Unstardardized Beta	*SE*	*p*	Standardized Beta
	Between-person effects			
Correlations				
Social Identity Threat → Sense of Belonging	-.21	.03	<.001	-.30
Social Identity Threat → Discussion Outdegree	-.01	.04	.772	-.02
Sense of Belonging → Discussion Outdegree	-0.006	.03	.843	-.01
	Within-person effects			
Auto-regressive paths				
Social Identity Threat T2 → Social Identity Threat T1	.21	.18	.241	.19

Sense of Belonging T2 → Sense of Belonging T1	.05	.14	.705	.06
Discussion Outdegree T2 → Discussion Outdegree T1	.66	.20	.001	.22
Social Identity Threat T3 → Social Identity Threat T2	.31	.11	.005	.33
Sense of Belonging T3 → Sense of Belonging T2	.27	.11	.014	.26
Discussion Outdegree T3 → Discussion Outdegree T2	.20	.05	<.001	.46
Cross-lagged paths				
Sense of Belonging T2 → Social Identity Threat T1	.18	.10	.065	.20
Discussion Outdegree T2 → Social Identity Threat T1	.16	.25	.515	.03
Discussion Outdegree T2 → Sense of Belonging T1[a]	.01	.25	.968	.002
Sense of Belonging T3 → Social Identity Threat T2	.07	.07	.265	.09
Discussion Outdegree T3 → Social Identity Threat T2	.11	.12	.385	.06
Discussion Outdegree T3 → Sense of Belonging T2	.09	.15	.580	.04
Discussion Outdegree T3 → Social Identity Threat T1	.09	.17	.600	.04

Note. [a] indicates a difference to the analyses with sense of belonging to CSCL group.

3 Supplement 3: Results of analyses regarding H3

Table S3

Results RI-CLPM H3 with sense of belonging to CSCL group

	Unstardardized Beta	*SE*	*p*	Standardized Beta
Between-person effects				
Correlations				
Social Identity Threat → Sense of Belonging	-.20	.03	< .001	-.40
Social Identity Threat → Social Approach Motivation	.02	.03	.458	.03
Sense of Belonging → Social Approach Motivation	.26	.04	< .001	.49
Within-person effects				
Auto-regressive paths				
Social Identity Threat T2 → Social Identity Threat T1	.16	.15	.293	.15
Sense of Belonging T2 → Sense of Belonging T1	.19	.13	.154	.19
Social Approach Motivation T2 → Social Approach Motivation T1	.04	.17	.816	.03
Social Identity Threat T3 → Social Identity Threat T2	.27	.11	.014	.28
Sense of Belonging T3 → Sense of Belonging T2	.47	.09	< .001	.48
Social Approach Motivation T3 → Social Approach Motivation T2	.32	.08	< .001	.31
Cross-lagged paths				
Sense of Belonging T2	.08	.11	.441	.06

→ Social Identity Threat T1				
Social Approach Motivation T2 → Social Identity Threat T1	.05	.11	.619	.05
Social Approach Motivation T2 → Sense of Belonging T1	-.04	.07	.573	-.05
Sense of Belonging T3 → Social Identity Threat T2	.17	.06	.008	.13
Social Approach Motivation T3 → Social Identity Threat T2	.01	.07	.879	.01
Social Approach Motivation T3 → Sense of Belonging T2	.03	.05	.584	.03
Social Approach Motivation T3 → Social Identity Threat T1	.15	.11	.156	-.13

4 Supplement 4: Results of analyses regarding H4

Table S4

Results RI-CLPM H4 with sense of belonging to CSCL group

	Unstardardized Beta	*SE*	*p*	Standardized Beta
	Between-person effects			
Correlations				
Social Identity Threat → Sense of Belonging	-.20	.03	< .001	-.41
Social Identity Threat → Discussion Outdegree	-.01	.04	.883	-.01
Sense of Belonging → Discussion Outdegree	.02	.03	.589	.05
	Within-person effects			

Auto-regressive paths				
Social Identity Threat T2 → Social Identity Threat T1	.21	.16	.186	.19
Sense of Belonging T2 → Sense of Belonging T1	.20	.14	.137	.20
Discussion Outdegree T2 → Social Approach Motivation T1	.65	.20	.001	.22
Social Identity Threat T3 → Social Identity Threat T2	.30	.11	.005	.32
Sense of Belonging T3 → Sense of Belonging T2	.49	.08	< .001	.49
Discussion Outdegree T3 → Social Approach Motivation T2	.21	.05	< .001	.47
Cross-lagged paths				
Sense of Belonging T2 → Social Identity Threat T1	.08	.10	.444	.06
Discussion Outdegree T2 → Social Identity Threat T1	.10	.24	.691	.02
Discussion Outdegree T2 → Sense of Belonging T1	-.35	.16	.030	-.10
Sense of Belonging T3 → Social Identity Threat T2	.15	.06	.009	.12
Discussion Outdegree T3 → Social Identity Threat T2	.13	.13	.336	.06
Social Approach Motivation T3 → Sense of Belonging T2	-.02	.08	.787	-.01
Discussion Outdegree T3 → Social Identity Threat T1	.11	.16	.485	.05

5 Supplement 5: Results of analyses controlling for CSCL group size

Table S5a

Results H1 with sense of belonging to CSCL group controlling for group size

		Direct Effects						
		Consequent						
		Sense of Belonging				Social Approach Motivation		
Antecedent		*b* [95% CI]	*SE*	*p*		*b* [95% CI]	*SE*	*p*
Social Identity Threat	a	-.22 [-.29; -.16]	.03	< .001	c	.09 [.02; .16]	.03	.007
Sense of Belonging		-	-	-	b	.35 [.29; .42]	.03	< .001
Group Size		.02 [-.03; .07]	.03	.523		-.05 [-.10; .00]	.02	.060
				Indirect Effect				
						b [95% CI]	*SE*	*p*
Social Identity Threat → Sense of Belonging → Social Approach Motivation					a*b	-.08 [-.11; -.05]	. 01	< .001

Table S5b

Results H1 with sense of belonging to university controlling for group size

		Direct Effects						
		Consequent						
		Sense of Belonging				Social Approach Motivation		
Antecedent		*b* [95% CI]	*SE*	*p*		*b* [95% CI]	*SE*	*p*
Social Identity Threat	a	-.22 [-.29; -.15]	.03	< .001	c	.09 [.03; .16]	.03	.007
Sense of Belonging		-	-	-	b	.36 [.29; .43]	.03	< .001
Group Size		-.02 [-.07; .03]	.03	.337		-.03 [-.08; .02]	.02	.193
				Indirect Effect				
						b [95% CI]	*SE*	*p*
Social Identity Threat → Sense of Belonging → Social Approach Motivation					a*b	-.08 [-.11; -.05]	. 01	< .001

Table S5c

Results H2 with sense of belonging to university controlling for group size

		Direct Effects										
		Consequent										
		Sense of Belonging				Social Approach Motivation				Behavioral Intentions		
Antecedent		*b* [95% CI]	*SE*	*p*		*b* [95% CI]	*SE*	*p*		*b* [95% CI]	*SE*	*p*
Social Identity Threat	a_1	-.22 [-.29; -.15]	.04	< .001	a_2	.09 [.02; .17]	.04	.018	c_p	.06 [.01; .11]	.03	.028
Sense of Belonging		-	-	-	d_{21}	.35 [.28; .42]	.04	< .001	b_1	.10 [.04; .16]	.03	< .001
Social Approach Motivation		-	-	-		-	-	-	b_2	.55 [.49; .60]	.03	< .001
Group Size		-.02 [-.07; .02]	.02	.324		-.03 [-.08; .02]	.02	.197		-.02 [-.05; .02]	.02	.345
		Indirect Effect										
										b [95%CI]	*SE*	*p*
Social Identity Threat → Social Approach Motivation → Sense of Belonging → Behavioral Intentions									$a_1*d_{12}*b_2$	-.04 [-.06; -.02]	.01	< .001

Table S5d

Results H2 with sense of belonging to CSCL group controlling for group size

		Direct Effects										
		Consequent										
		Sense of Belonging				Social Approach Motivation				Behavioral Intentions		
Antecedent		*b* [95% CI]	*SE*	*p*		*b* [95% CI]	*SE*	*p*		*b* [95% CI]	*SE*	*p*
Social Identity Threat	a_1	-.22 [-.29; -.15]	.03	< .001	a_2	.09 [.02; .15]	.03	.007	c_p	.04 [.01; .10]	.03	.100
Sense of Belonging		-	-	-	d_{21}	.35 [.28; .41]	.03	< .001	b_1	.03 [-.02; .10]	.03	.268

Social Approach Motivation	-	-	-	-	-	-	b_2	.57 [.52; .62]	.03	< .001
Group Size	.01 [-.03; .07]	.03	.523	-.05[-.09; .00]	.02	.061		-.02 [-.06; .02]	.02	.326
				Indirect Effect						
								b [95%CI]	*SE*	*p*
Social Identity Threat → Social Approach Motivation → Sense of Belonging → Behavioral Intentions							$a_1*d_{12}*b_2$	-.04 [-.06; -.03]	.01	< .001

6 Supplement 6: Frequencies of group identification per measurement occasion

Table S6

Absolute (n) and relative (%) frequencies for each student group and measurement occasion

	T0		T1		T2		T3	
Group Identification	*n*	%	*n*	%	*n*	%	*n*	%
Female Students	450	37.19	574	47.44	657	54.30	577	47.69
Male Students	201	16.61	196	16.20	211	17.44	209	17.27
Students with Chronic Illness	78	6.45	119	9.83	119	9.83	98	8.10
Students with Disability	22	1.82	36	2.98	34	2.81	31	2.56
Students with Children	121	10.00	154	12.73	178	14.71	166	13.72
Full-Time Employed Students	451	37.27	395	32.64	416	34.38	369	30.50
Older Students	188	15.54	354	29.26	370	30.58	342	28.26
Younger Students	-	-	362	29.92	406	33.55	323	26.69
Students with Migration Background	92	7.60	167	13.80	180	14.88	162	13.39
Students with non-German Native Language	56	4.63	94	7.77	97	8.02	84	6.94

Note. Please note that due to programming issues, the group younger students was not assessed at T0.

7 Supplement 7: Results of analyses controlling for off-system behavior

Table S7a

Results H1 with sense of belonging to CSCL group controlling for off-system behavior

		Direct Effects						
		Consequent						
		Sense of Belonging				Social Approach Motivation		
Antecedent		*b* [95% CI]	*SE*	*p*		*b* [95% CI]	*SE*	*p*
Social Identity Threat	a	-.22 [-.28; -.15]	.03	< .001	c	.09 [.02; .16]	.03	.007
Sense of Belonging		-	-	-	b	.33 [.27; .40]	.03	< .001
Off-System Behavior		.14 [.08; .20]	.03	< .001		.10 [.04; .16]	.03	.001
				Indirect Effect				
						b [95% CI]	*SE*	*p*
Social Identity Threat → Sense of Belonging → Social Approach Motivation					a*b	-.07 [-.10; -.05]	. 01	< .001

Table S7b

Results H1 with sense of belonging to university controlling for off-system behavior

		Direct Effects						
		Consequent						
		Sense of Belonging				Social Approach Motivation		
Antecedent		*b* [95% CI]	*SE*	*p*		*b* [95% CI]	*SE*	*p*
Social Identity Threat	a	-.22 [-.29; -.15]	.03	< .001	c	.10 [.03; .16]	.03	.003
Sense of Belonging		-	-	-	b	.36 [.29; .43]	.03	< .001
Off-System Behavior		-.02 [-.08; .04]	.03	.579		.15 [.10; .21]	.03	< .001
				Indirect Effect				
						b [95% CI]	*SE*	*p*
Social Identity Threat → Sense of Belonging → Social Approach Motivation					a*b	-.08 [-.11; -.05]	. 01	< .001

Table S7c

Results H2 with sense of belonging to university controlling for off-system behavior

		Direct Effects										
		Consequent										
		Sense of Belonging				Social Approach Motivation				Behavioral Intentions		
Antecedent		*b* [95% CI]	*SE*	*p*		*b* [95% CI]	*SE*	*p*		*b* [95% CI]	*SE*	*p*
Social Identity Threat	a_1	-.22 [-.29; -.15]	.03	< .001	a_2	.09 [.03; .16]	.03	.003	c_p	.06 [.01; .11]	.03	.028
Sense of Belonging		-	-	-	d_{21}	.36 [.29; .42]	.03	< .001	b_1	.10 [.05; .15]	.03	< .001
Social Approach Motivation		-	-	-		-	-	-	b_2	.55 [.49; .60]	.03	< .001
Off-System Behavior		-.02 [-.08; .04]	.02	.579		-.03 [-.08; .02]	.02	.197		.01 [-.03; .06]	.02	.629
						Indirect Effect						
										b [95%CI]	*SE*	*p*
Social Identity Threat → Social Approach Motivation → Sense of Belonging → Behavioral Intentions									$a_1*d_{12}*b_2$	-.04 [-.06; -.03]	.01	< .001

Table S7d

Results H2 with sense of belonging to CSCL group controlling for off-system behavior

		Direct Effects										
		Consequent										
		Sense of Belonging				Social Approach Motivation				Behavioral Intentions		
Antecedent		*b* [95% CI]	*SE*	*p*		*b* [95% CI]	*SE*	*p*		*b* [95% CI]	*SE*	*p*
Social Identity Threat	a_1	-.22 [-.29; -.15]	.03	< .001	a_2	.09 [.02; .15]	.03	.008	c_p	.04 [.01; .10]	.03	.100
Sense of Belonging		-	-	-	d_{21}	.33 [.26; .39]	.03	< .001	b_1	.03 [-.02; .08]	.03	.288

Social Approach Motivation	-	-	-	-	-	-	b_2	.57 [.52; .62]	.03	< .001
Off-System Behavior	.14 [.08; .20]	.03	< .001	.10[.04; .16]	.03	.001		.001 [-.04; .05]	.02	.949
				Indirect Effect						
								b [95%CI]	*SE*	*p*
Social Identity Threat → Social Approach Motivation → Sense of Belonging → Behavioral Intentions							a_1*d_{12}*b_2	-.04 [-.06; -.03]	.01	< .001
